# Identification of key molecular targets in nicotine-induced spontaneous abortion through network toxicology and multi-omics integration

**DOI:** 10.18332/tid/230997

**Published:** 2026-09-22

**Authors:** Kaiyan Yang, Xinying Song, Pengyan Zhai, Huiyan Wang, Wenbo Zhou

**Affiliations:** 1 Changzhou Maternal and Child Health Care Hospital, Changzhou Medical Center, Nanjing Medical University, Changzhou, China

**Keywords:** nicotine, spontaneous abortion, network toxicology, multi-omics, molecular docking

## Abstract

**INTRODUCTION:**

Spontaneous abortion (SA) remains a prevalent reproductive health challenge, with tobacco-derived nicotine emerging as a significant risk factor. This study sought to decipher the molecular underpinnings of nicotine-induced pregnancy loss through comprehensive multi-omics profiling to identify novel biomarkers and potential intervention targets.

**METHODS:**

An integrated bioinformatics approach was implemented combining network toxicology, transcriptomic profiling, and machine learning algorithms to elucidate nicotine's pathological mechanisms in SA. The analytical pipeline encompassed functional enrichment analysis, expression pattern characterization, diagnostic biomarker evaluation, immune microenvironment assessment, causal inference through Mendelian randomization (MR), single-cell RNA sequencing, and molecular docking simulations of nicotine-protein interactions.

**RESULTS:**

Differential expression analysis identified 2451 DEGs between SA and control groups. Integration with 645 nicotine targets yielded 69 candidate genes. Five hub genes – CTNNB1, TINAGL1, RAD50, FGF2, and VIM – were identified as critical mediators in nicotine-triggered SA pathogenesis. These genes exhibited good diagnostic capability and significant correlations with immune cell infiltration patterns. MR analysis confirmed causal associations for RAD50 (OR=1.08; 95% CI: 1.01–1.15, p=0.019) and FGF2 (OR=1.21; 95% CI: 1.11–1.33, p=4.69×10^-5^). Single-cell analyses revealed cell-type-specific expression signatures across trophoblasts and immune populations, while molecular docking showed binding energies ranging from -5.0 to -6.9 kcal/mol.

**CONCLUSIONS:**

This study illuminates a complex, multi-target mechanism underlying tobacco-induced pregnancy loss. This work advances understanding of nicotine's reproductive toxicity pathways and identifies promising molecular targets for early diagnosis and further mechanistic studies.

## INTRODUCTION

Spontaneous abortion (SA) refers to pregnancy loss occurring before 20 weeks of gestation or when the fetal weight is <500 g, and it is one of the most common complications during early pregnancy^1^. According to recent epidemiological surveys, approximately 15–20% of clinically recognized pregnancies end in SA; when including undetected biochemical pregnancies, the overall incidence may reach as high as 30–50%^1^. SA not only leads to severe physiological complications such as hemorrhage and infection in women but also frequently triggers psychological disorders such as anxiety and depression, imposing substantial physical, emotional, and economic burdens on individuals, families, and society^2^. Although more than half of early SA can be attributed to genetic abnormalities such as embryonic chromosomal aneuploidy, nearly half of cases remain idiopathic, suggesting a significant role of modifiable factors such as environmental exposures and lifestyle in the etiology of SA^3^.

Among various modifiable environmental factors, tobacco exposure is widely recognized as one of the most prominent risk factors affecting reproductive health in women of reproductive age^4^. Numerous prospective cohort studies and case-control studies have consistently confirmed a significant association between active or passive smoking during pregnancy and increased risk of spontaneous abortion^5^.

Nicotine, the most abundant addictive alkaloid in tobacco, is considered the primary bioactive component mediating smoking-related reproductive toxicity. Studies have shown that nicotine can rapidly cross the placental barrier and enter the fetal circulation, with concentrations in amniotic fluid, fetal blood, and tissues even exceeding those in maternal plasma^6^. Known mechanisms by which nicotine contributes to miscarriage involve multiple biological levels: inducing vasoconstriction of uterine and placental arteries via activation of the sympathetic nervous system, leading to impaired placental perfusion and fetal hypoxia; triggering oxidative stress and endoplasmic reticulum stress in placental trophoblast cells, thereby compromising placental barrier integrity; and modulating decidual immune cell function, disrupting maternal-fetal immune tolerance^7^.

Although these studies provide important biological evidence for nicotine’s reproductive toxicity, significant knowledge gaps remain. First, the precise molecular targets and key signaling pathways through which nicotine acts at the maternal-fetal interface have not been fully elucidated. Second, the differential sensitivity of various cell types to nicotine and their specific regulatory networks remain unclear. Network toxicology, an emerging interdisciplinary field integrating systems biology and toxicology, enables the systematic elucidation of the toxicity mechanisms of exogenous chemicals by constructing multidimensional interaction networks among ‘compounds, targets, and diseases’^8^. Mendelian randomization (MR) analysis, an innovative epidemiological method, uses genetic variants as instrumental variables to infer causal relationships between exposures and disease outcomes, effectively minimizing confounding and reverse causality^9^. The rapid advancement of single-cell RNA sequencing (scRNA-seq) technology has provided a revolutionary tool for dissecting cellular heterogeneity and molecular mechanisms in pregnancy-related conditions^10^. Molecular docking, based on structural biology and computational chemistry, simulates the three-dimensional binding modes between small molecule ligands and target proteins, quantitatively evaluating the affinity and specificity of molecular interactions^11^.

This study establishes a systematic research framework integrating network toxicology prediction, transcriptomic differential analysis, single-cell sequencing validation, Mendelian randomization for causal inference, and molecular docking simulation to systematically identify key molecular targets of nicotine associated with spontaneous abortion and elucidate their expression profiles and regulatory networks across different cell types at the maternal-fetal interface.

## METHODS

### Identification of potential targets for nicotine

We systematically identified potential targets of nicotine by integrating multiple authoritative drug target prediction databases: 1) SwissTargetPrediction database with species set to ‘Homo sapiens’ and predicted targets with probability values ≥0.1; 2) STITCH compound-protein interaction database using a confidence score threshold of ≥0.4; 3) DGIdb drug-gene interaction database to collect reported nicotine interaction targets; and 4) Comparative Toxicogenomics Database screened for interaction targets supported by at least one peer-reviewed publication.

### Transcriptomic differential expression analysis and biomarker screening

We analyzed the endometrial tissue transcriptomic dataset GSE165004 from the GEO database, comprising decidual tissue samples from 24 recurrent SA patients and 24 normal early pregnancy women. After quality control, we performed normalization and differential expression analysis using the *limma* package in R. Differentially expressed genes (DEGs) were screened using adjusted p<0.05. Subsequently, intersection analysis was performed between DEGs and the nicotine target gene set.

### Functional enrichment and pathway analysis

Multi-dimensional functional annotation and pathway enrichment analyses were performed on the intersected gene set to elucidate the underlying molecular mechanisms. Gene Ontology (GO) analysis systematically examined gene functional hierarchies from three perspectives: biological process, molecular function, and cellular component. Kyoto Encyclopedia of Genes and Genomes (KEGG) pathway enrichment analysis focused on revealing signal transduction networks and metabolic pathways involving these genes. All enrichment analyses were implemented using the *clusterProfiler* R package^12^, with significance levels set at p<0.05 (FDR corrected).

### Machine learning algorithms for key gene identification

To identify core genes with the highest discriminative power from high-dimensional gene lists, this study employed two complementary machine learning feature selection methods: LASSO regression and Support Vector Machine-Recursive Feature Elimination (SVM-RFE). LASSO regression analysis was implemented using the *glmnet* R package^13^, constructing an L1-regularized logistic regression model with group labels as response variables and intersection gene expression profiles as predictor variables. The optimal penalty parameter λ (lambda. min) was determined through 10-fold cross-validation, and genes corresponding to non-zero coefficients were retained as first-tier screening results. SVM-RFE analysis was performed using the *e1071* and *caret* R packages^14^, constructing support vector machine classifiers and iteratively removing features with the smallest weights until the remaining feature set achieved minimal classification error. Finally, the intersection of genes from both methods was taken as the final core signature gene set to enhance the robustness and generalizability of the screening results.

### Expression profiling and diagnostic evaluation of key genes

Expression level visualization and diagnostic efficacy analysis were performed on the screened core genes within the GSE165004 dataset. Box plots were generated using the *ggplot2* package to intuitively display expression difference patterns of each gene between recurrent SA and NEP groups. Simultaneously, receiver operating characteristic (ROC) curves and their corresponding areas under the curve (AUC) were calculated for each core gene to quantitatively assess their diagnostic potential as biomarkers.

### Immune infiltration analysis

The CIBERSORTx algorithm was employed to perform deconvolution analysis on gene expression profiles from GSE165004, estimating the relative abundance of 22 immune cell subsets^15^. The analysis used limma-normalized expression matrices as input data, with Leukocyte Gene Signature Matrix (LM22) selected as the reference signature matrix. Through Spearman rank correlation analysis, we systematically calculated correlation coefficients between core gene expression levels and the abundance of various immune cells, and visualized the gene-immune cell association network in heatmap format. Wilcoxon rank-sum tests were used to compare differences in immune cell composition between groups to reveal potential immune imbalance mechanisms.

### MR analysis

To assess the potential causal association between identified key target genes and SA, we employed a two-sample Mendelian randomization (MR) design. The validity of this approach relies on three core assumptions: 1) the relevance assumption, requiring that the genetic instruments are strongly associated with the exposure; 2) the independence assumption, requiring that the instruments are not associated with confounders of the exposure-outcome relationship; and 3) the exclusion restriction assumption, requiring that the instruments influence the outcome only through the exposure, not via alternative pathways.

To satisfy the relevance assumption, instrumental variables (IVs) were selected from expression quantitative trait loci (eQTL) data at a genome-wide significance threshold (p<5×10^-8^). Only single nucleotide polymorphisms (SNPs) with F-statistic >10 were retained, ensuring that the instruments were sufficiently strong and effectively minimizing weak instrument bias. When a selected IV was not present in the outcome data, it was excluded from the MR analysis. For the independence assumption, we obtained eQTL summary statistics from the IEU Open GWAS resource, which derives primarily from the eQTLGen consortium (n=31684 individuals)^16^, while outcome data for SA were drawn from the FinnGen R12 dataset (23167 cases and 199279 controls), an independent Finnish population cohort^17^ (Supplementary file Table 1). Both the exposure and outcome GWAS were derived from European-ancestry populations and are independent, with no sample overlap. Furthermore, linkage disequilibrium (LD) clumping was performed (r^2^<0.1, window size 10000 kb) to ensure that the selected SNPs were independent of one another. Target genes for which no eQTLs were available in the eQTLGen resource, or for which no instrumental variable passed the selection criteria, were excluded from causal estimation.

The primary causal effect was estimated using the inverse variance weighted (IVW) method. To thoroughly assess the exclusion restriction assumption, multiple supplementary analyses were implemented to detect and adjust for potential horizontal pleiotropy. Specifically, we applied the weighted median and MR-Egger regression methods, which are more robust to pleiotropy than the IVW approach under different scenarios. Heterogeneity among the IVs was evaluated with Cochran’s Q test, while the MR-Egger intercept test was used as a formal test of directional pleiotropy. In addition, the MR-PRESSO global test was employed to identify outlier SNPs that might violate the exclusion restriction. Leave-one-out analyses were further conducted to confirm that the overall causal estimates were not driven by any single variant.

### Single-cell validation of core target gene expression

To validate the expression characteristics of the aforementioned core target genes in recurrent abortion at single-cell resolution, we analyzed the single-cell transcriptomic dataset GSE214607 from the GEO database, which encompassed decidual tissue samples from 3 SA patients and 5 control women. The original gene-cell expression matrix was generated using the 10x Genomics platform and processed using the *Seurat* package in R. Quality control was first performed on cells, and potential doublets were removed using *DoubletFinder*. After using *LogNormalize*, highly variable genes were selected and the *Harmony* algorithm was employed to correct for sample batch effects. Based on t-SNE dimensionality reduction clustering, cell type annotation was performed according to marker genes from reference literature. Subsequently, the core target genes identified in the previous study were mapped to various cell types, and their expression distribution characteristics were visualized through dot plots and feature plots. Wilcoxon rank-sum tests were applied to compare expression differences of target genes in each cell type between SA and control groups, with results displayed through violin plots.

### Molecular docking validation

To predict the binding capacity between nicotine and core target proteins, this study employed the CB-Dock2 online server for molecular docking analysis. CB-Dock2 integrates the curvature-driven binding site identification algorithm with the AutoDock Vina docking engine, enabling efficient prediction of receptor protein binding cavities and ligand docking simulation^18^. Three-dimensional structures of core target proteins were obtained from the Protein Data Bank. For proteins lacking experimentally resolved structures, high-precision structural models predicted by AlphaFold2 were employed. The three-dimensional structure of nicotine was retrieved from the PubChem database (CID: 89594). Docking parameters were set to program defaults, binding energy scoring employed the Vina scoring function, and the highest affinity conformation and predicted binding free energy (kcal/mol) for each complex were systematically recorded.

### Statistical analysis

All bioinformatics and statistical analyses were performed using R software (version 4.3.2). Differential expression analysis was conducted with the *limma* package, applying the Benjamini-Hochberg procedure for multiple testing correction (adjusted p<0.05). Diagnostic performance was evaluated by receiver operating characteristic (ROC) curves, with an area under the curve (AUC) >0.7 considered indicative of acceptable discriminatory ability. For immune infiltration comparisons, Wilcoxon rank-sum tests were used for between-group comparisons, and Spearman’s rank correlation was used for association analyses. MR analyses were performed using the *TwoSampleMR* (version 0.6.8) and *MR-PRESSO* (version 1.0) packages, the IVW method served as the primary estimator, with a p<0.05 considered statistically significant. Heterogeneity was evaluated with Cochran’s Q test, and horizontal pleiotropy was assessed using the MR-Egger intercept test; for both tests, p<0.05 was interpreted as evidence of significant heterogeneity or pleiotropy, respectively. Molecular docking was carried out using CB-Dock2, and binding energies ≤ -5.0 kcal/mol were considered indicative of significant binding affinity.

## RESULTS

### Network toxicology prediction of key targets in nicotine-induced SA

Integration of data from SwissTargetPrediction, STITCH, DGIdb, and CTD databases yielded 645 potential nicotine targets (Supplementary file Figure 1). Differential expression analysis of GEO datasets revealed 2451 DEGs, visualized through volcano plots and heatmaps (Supplementary file Figures 2 and 3). We identified 69 overlapping genes between nicotine targets and SA-related genes (Supplementary file Figure 4), which were used to construct a protein-protein interaction network (Supplementary file Figure 5).

GO enrichment analysis revealed that these targets were primarily involved in hormone response (glucocorticoid and steroid), apoptosis regulation, and mechanical stimulus response. Cellular components were enriched in outer membranes, membrane rafts, and synaptic structures, while molecular functions concentrated on ubiquitin ligase activity, phosphatase activity, and protein binding (Supplementary file Figure 6). KEGG pathway analysis demonstrated significant enrichment in viral infection pathways, cancer-related pathways, apoptotic pathways, and the PD-L1 pathway (Supplementary file Figure 7).

### Machine learning identification of key diagnostic genes

Using the 69 candidate target genes, we employed complementary machine learning approaches for feature selection. LASSO regression identified 15 genes with non-zero coefficients (Supplementary file Figures 8 and 9), while SVM-RFE algorithm indicated an optimal feature number of 5 (Supplementary file Figure 10). Intersection analysis revealed five key genes: CTNNB1, FGF2, RAD50, TINAGL1, and VIM (Supplementary file Figure 11).

Expression analysis demonstrated significant differences between SA and control groups for all five genes: TINAGL1 was upregulated in SA, while CTNNB1, FGF2, RAD50, and VIM were downregulated (Supplementary file Figure 12). ROC curve analysis validated the diagnostic efficacy of all five genes, with AUC values ranging from 0.747 to 0.969 (Supplementary file Figure 13).

### Immune infiltration analysis

**Figure 1:**
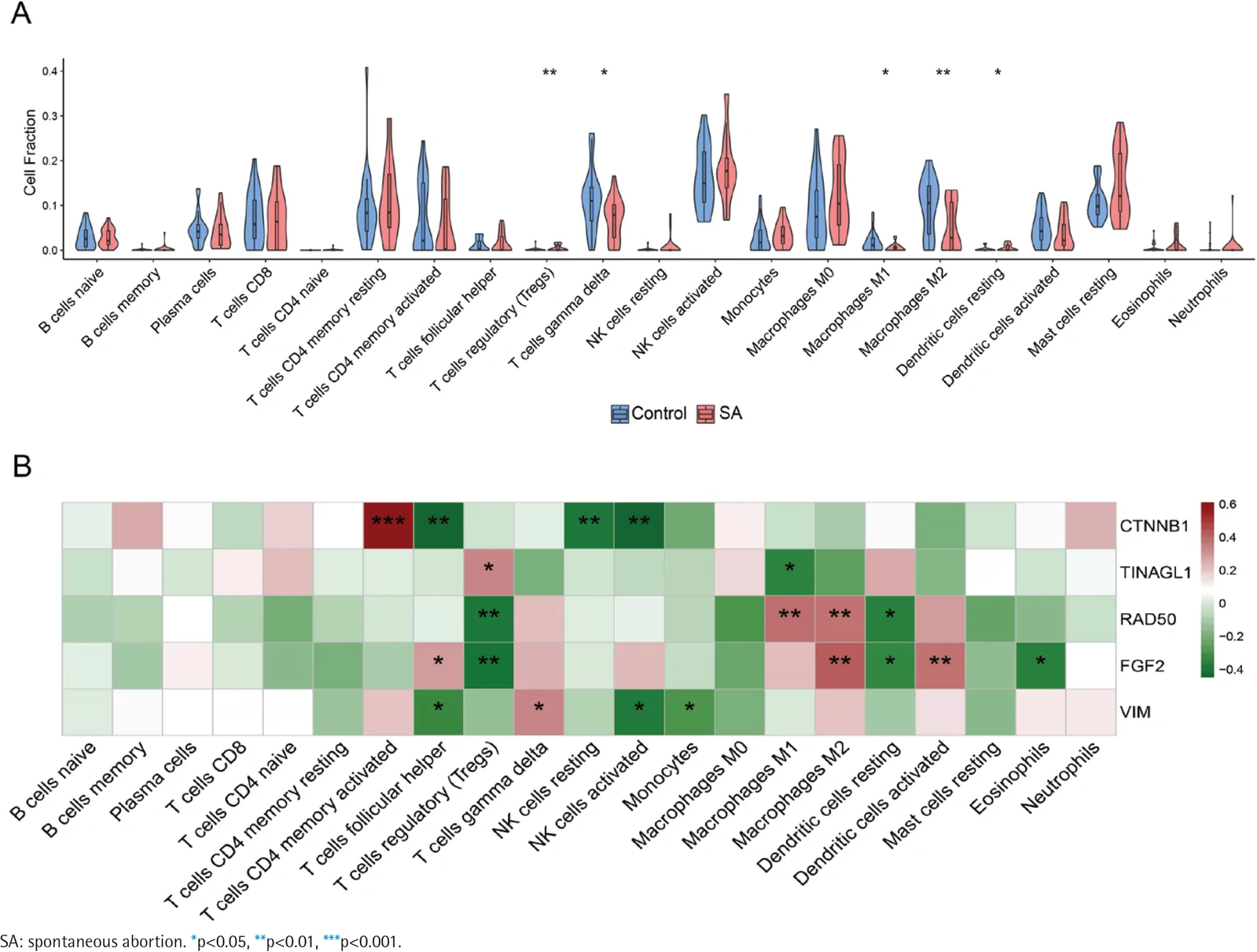
Immune infiltration analysis: A) Violin plots comparing the distributions of each immune-cell subset between control and SA samples; B) Spearman correlation heatmap between the infiltration levels of the immune-cell subsets and the five hub genes

The distribution of 22 immune cell types revealed distinct compositional patterns between groups (Supplementary file Figure 14), with activated mast cells showing no infiltration signal across all samples. Correlation analysis revealed complex intercellular interactions, such as the positive correlation between M2 macrophages and plasma cells and the associations among other immune subsets (Supplementary file Figure 15). The SA group exhibited significant alterations in regulatory T cells, γδ T cells, macrophages (M1 and M2), and dendritic cell proportions (Figure 1A).

Correlation analysis between key genes and immune cell infiltration showed that CTNNB1 positively correlated with activated memory CD4+ T cells while negatively correlating with follicular helper T cells and NK cells. RAD50 significantly correlated with regulatory T cells, macrophages, and dendritic cells. FGF2 positively correlated with M2 macrophages and activated dendritic cells but negatively with regulatory T cells. Both TINAGL1 and VIM were associated with specific T cell subsets and myeloid cells (Figure 1B).

### MR analysis validates causal associations

Since no expression-associated signals for TINAGL1 were found in the eQTLGen database, we employed two-sample MR to evaluate causal associations between four potential target genes (CTNNB1, RAD50, FGF2, and VIM) and SA. The IVs are shown in Supplementary file Table 2, with the F-statistic ranging from 30.58 to 1140.08, indicating no evidence of weak instrument bias.

The MR analysis results showed significant causal associations between FGF2 (95% CI: 1.11–1.33, p=4.69×10^-5^) and RAD50 (OR=1.08; 95% CI: 1.01– 1.15, p=0.019) and SA risk (Supplementary file: Table 3 and Figure 16). Sensitivity analyses further supported the robustness of these findings (MR-Egger intercept p>0.05) (Supplementary file Tables 3 and 4).

### Single-cell transcriptome analysis validates cell-specific expression of core genes

After quality control and standardization, we obtained 48051 high-quality cells, including 20799 cells from the SA group and 27252 cells from the control group. Through t-SNE dimensionality reduction combined with cell type marker genes indicated by the data source references^19^, we identified 11 cell types consistent with those reported in the literature, including B cells, T cells, dendritic cells (DCs), decidual NK cells (dNKs), decidual macrophages (dM), monocytes (Mon), neutrophils, decidual stromal cells (DSCs), endothelial cells (Endo), epithelial cells (Epi), and extravillous trophoblasts (EVT).

Dot plot and feature plot analyses revealed distinct cell type-specific expression patterns of the five core genes (Supplementary file Figures 18 and 19). VIM showed high expression in stromal-derived and immune cells, particularly in DSCs, dM, and DCs. CTNNB1 was enriched in tissue barrier-related cells (Endo, DSCs, EVT) and specific immune subsets. TINAGL1 exhibited highly tissue-specific expression, primarily in EVT and Endo cells. FGF2 maintained low basal expression across most cell types, with high expression only in DSCs. RAD50 showed relatively uniform expression across all cell populations, with slight enrichment in EVT and DSCs.

Differential expression analysis revealed significant cell type-specific changes (Supplementary file Figure 20). TINAGL1 was significantly upregulated across multiple cell types (Epi, DSCs, dNK, dM, and EVT). CTNNB1 was significantly downregulated in EVT, Mon, dNK cells, and DSCs, but upregulated in Epi. RAD50 was significantly downregulated in DSCs, Mon, and Endo, but upregulated in EVT and Epi. FGF2 was significantly downregulated in DSCs, dM, EVT, and Mon, showing upregulation trends only in T cells. VIM was significantly upregulated across multiple cell types including Epi, dNK, dM, DCs, T cells, and neutrophils.

### Molecular docking analysis

Crystal structures of four target proteins were obtained from the PDB: β-catenin (CTNNB1, ID: 1JDH), RAD50 (ID: 5GOX), FGF2 (ID: 1CVS), and VIM (ID: 3KLT). For TINAGL1, we utilized its AlphaFold structure (ID: AF-Q9GZM7-F1).

Nicotine formed stable ligand-protein complexes with all five target proteins (Supplementary file Figure 21), with binding free energies ranging from -5.0 to -6.9 kcal/mol, consistent with moderate to high-affinity small molecule-protein interactions. Notably, nicotine formed the most stable complex with cytoskeletal protein VIM (-6.9 kcal/mol).

## DISCUSSION

This study employed an integrated multi-omics approach to systematically elucidate the molecular mechanisms by which nicotine may contribute to SA through multi-target, multi-pathway, and cell-specific regulatory networks. Through the integration of network pharmacology, transcriptomics, single-cell genomics, and molecular docking techniques, this study identified five core targets and demonstrated their aberrant expression in SA and associations with immune microenvironmental alterations, establishing a novel framework for understanding nicotine’s pathological effects on pregnancy maintenance.

Network pharmacology analysis revealed that common targets between nicotine and SA were primarily enriched in hormonal response regulation, apoptotic pathways, and immune checkpoint processes. These findings align with previous studies demonstrating nicotine’s effects on hormonal balance, cellular apoptosis, and immune microenvironment modulation during pregnancy^20^. The enrichment of viral infection-related pathways is particularly intriguing, as emerging evidence suggests that disruption of antiviral immune pathways may compromise placental immune surveillance mechanisms^21^. Recent research has demonstrated that placental tissues express numerous antiviral response genes that not only protect against viral pathogens but also regulate trophoblast invasion and differentiation, suggesting potential convergence between immune defense mechanisms and pregnancy maintenance pathways that may be disrupted by nicotine exposure^22^.

The five core target genes identified through integrated machine learning approaches all demonstrated substantial diagnostic value with clear biological plausibility for involvement in pregnancy maintenance. CTNNB1, encoding β-catenin as the core transducer of Wnt signaling, plays a pivotal role in embryonic implantation and placental formation^23^. The single-cell analysis revealed CTNNB1 expression across multiple placental cell types, particularly abundant in Endo, DSCs, and EVT, but significantly downregulated in DSCs, dNK, and EVT in abortion samples. This observation aligns with previous studies demonstrating associations between aberrant Wnt/β-catenin signaling and increased abortion risk^23,24^.

TINAGL1 exhibited remarkable diagnostic value, with significantly elevated expression in decidual tissues from abortion patients. As an extracellular matrix protein involved in cellular adhesion, migration, and angiogenesis^25^, its role as a positive regulator of angiogenesis and expression in mouse uterine decidua following implantation is particularly relevant, considering that insufficient trophoblast invasion constitutes an important pathological basis for abortion. Single-cell analysis demonstrated that TINAGL1 is primarily expressed in EVT and endothelial cells, both crucial for placental vascular remodeling. Previous studies have shown that TINAGL1 functions as an integrin ligand involved in endometrial stromal cell adhesion during embryo implantation^26^ and co-localizes with extracellular matrix proteins during pre-implantation embryonic development^27^. It is hypothesized that TINAGL1 upregulation in abortion patients may represent a pathological adaptive response, potentially reflecting a compensatory mechanism attempting to repair damaged maternal-fetal interfaces, though this excessive response may ultimately disrupt normal extracellular matrix balance.

FGF2 demonstrated strong causal evidence for association with SA in MR analysis, aligning with previous studies showing that nicotine exposure alters FGF2 gene and protein expression and impair FGF2 signaling systems^28^. As a crucial regulator of angiogenesis and tissue repair, FGF2 promotes placental vascular network formation and decidualization during normal pregnancy^29^. The single-cell analysis revealed significant FGF2 downregulation in decidual stromal cells from abortion patients, consistent with reduced FGF2 expression observed in placental tissues from women with recurrent pregnancy loss. The significant positive correlation between FGF2 and M2 macrophage abundance supports its involvement in maternal-fetal interface immune regulation, aligning with previous reports of FGF2’s role in promoting M2 macrophage polarization^30^. Given that M2 macrophages play protective roles in maintaining maternal-fetal immune tolerance^31^, these findings provide new perspectives for understanding how environmental factors like smoking affect pregnancy outcomes through specific molecular pathways.

RAD50, a core component of the DNA double-strand break repair complex^32^, showed complex regulatory patterns in the analyses. While MR analysis suggested that increased RAD50 in blood correlates positively with abortion risk, the transcriptomic data revealed significant downregulation in abortion decidual tissue, with single-cell analysis uncovering complex cell type-specific expression patterns. Given that smoking causes DNA damage^33^, and that DNA repair capacity is crucial during early embryonic development, it is hypothesized that nicotine may interfere with RAD50-mediated DNA repair function, leading to increased genomic instability in placental cells. The correlation between RAD50 and regulatory T cells/macrophages abundance is consistent with emerging research proposing crossover mechanisms between DNA damage response and immune regulation^34^.

VIM demonstrated the highest binding affinity with nicotine in molecular docking analysis, suggesting it may represent an important direct target. As an intermediate filament protein involved in cytoskeletal organization and epithelial-mesenchymal transition (EMT) processes^35^, VIM showed significant upregulation across multiple immune cell types in SA patients, consistent with its functions in immune cell activation^36^.

### Limitations

While the integration of network toxicology with multi-omics data provides novel insights into environmentally induced pregnancy loss mechanisms, several methodological limitations warrant consideration. These include: the necessity for experimental validation of predicted nicotine-protein interactions; limited representativeness due to restricted decidual tissue samples for single-cell sequencing; interpretive constraints of the MR analyses, which reflect cumulative rather than stage-specific genetic effects, and the use of data from predominantly European participants, potentially limiting the generalizability of the findings to other populations; the potential for residual confounding in the transcriptomic and immune infiltration analyses, as they are based on observational data; and the inherent limitations of bulk transcriptomics in capturing cell-specific alterations. These constraints highlight critical directions for future investigations.

## CONCLUSIONS

Through network toxicology and multi-omics integration, this study elucidated the molecular mechanisms underlying nicotine-induced spontaneous abortion (SA) and identified several key target genes. These findings advance the understanding of how environmental exposures disrupt maternal-fetal homeostasis and provide a molecular framework for biomarker-based risk prediction and further mechanistic studies.

## Supplementary Material



## Data Availability

The transcriptomic (GSE165004) and single-cell (GSE214607) datasets are publicly available in the Gene Expression Omnibus (GEO; https://www.ncbi.nlm.nih.gov/geo/). Summary statistics for Mendelian randomization were sourced from the IEU Open GWAS project (https://gwas.mrcieu.ac.uk/) and the FinnGen R12 release (https://www.finngen.fi/en/access_results). Other web resources used include SwissTargetPrediction (http://www.swisstargetprediction.ch/), STITCH (http://stitch.embl.de/), DGIdb (https://www.dgidb.org/), CTD (http://ctdbase.org/), PDB (https://www.rcsb.org/), UniProt (https://www.uniprot.org/), PubChem (https://pubchem.ncbi.nlm.nih.gov/), and the CB-Dock2 server (https://cadd.labshare.cn/cb-dock2/). The data that support the findings of this study are available within the article or its Supplementary file. Further inquiries can be directed to the corresponding author.
