# Supplementary material for: Identification of key molecular targets in nicotine-induced spontaneous abortion through network toxicology and multi-omics integration
Source: Tob Induc Dis. 2026 Sep 22;24:10.18332/tid/230997. doi: 10.18332/tid/230997 (PMC13596037; doi:10.18332/tid/230997)

TID
Tobacco Induced Diseases

Supplementary file

© 2026 Yang K. et al.

DOI:
10.18332/tid/230997

The content has been provided by the author(s) and has not been reviewed, verified, or endorsed by European Publishing. It may not have undergone peer review. The views, opinions, and recommendations expressed are solely those of the author(s) and do not necessarily reflect the position of European Publishing. European Publishing accepts no responsibility or liability for any consequences arising from the use of, or reliance on, this content.

Supplementary Table S1. Sources of data for Mendelian randomization analysis

Item	Exposure data (eQTLGen)	Outcome data (Spontaneous abortion)
Data source	eQTLGen Consortium (phase I)	FinnGen Release 12 (R12:O15_ABORT_SPONTAN)
Primary publication	Võsa U et al. Nat Genet. (PMID: 34475573)	Kurki MI et al. Nature. (PMID: 36653562)
Setting	37 multinational cohorts (Netherlands, Estonia, Finland, Germany, UK, Italy, US, and others	Nationwide population-based project in Finland
Participants	31,684 individuals of European ancestry	23,167 cases and 199,279 controls from biobank participants; European ancestry
Sample type	Whole blood and PBMC	Whole blood
Eligibility criteria	Blood-derived eQTL cohorts with available genotype and expression data; unrelated individuals (except FHS)	Biobank donors with informed consent for biobank research per Finnish Biobank Act; includes legacy and prospective samples
Genotyping platform	Illumina and Affymetrix arrays (cohort-specific); imputation to 1000 Genomes phase 1 v3	Illumina and Affymetrix arrays; imputation to population-specific SISu v.3 reference panel
Outcome assessment	N/A (exposure data only)	Identified from Finnish national health registers (ICD codes)
Ethics approval	Each cohort obtained local IRB approval; details in original publications	Coordinating Ethics Committee of the Hospital District of Helsinki and Uusimaa (HUS/990/2017)
Informed consent	Written informed consent obtained from all participants	Participants provided informed consent for biobank research based on the Finnish Biobank Act

Supplementary Table S2. Information on genetic instrumental variables

	SNP	A1	A2	beta	se	P	F
CTNNB1	rs1722846	C	T	0.389	0.012	1.00E-200	1140.08
	rs12492719	C	A	0.671	0.028	8.00E-131	592.28
	rs422711	A	G	-0.164	0.012	1.57E-43	191.4
	rs11564445	T	C	-0.312	0.026	5.40E-34	147.74
	rs62259308	C	G	-0.163	0.014	1.13E-32	141.7
	rs436912	T	C	0.151	0.013	1.19E-29	127.89
	rs6777649	A	G	0.157	0.017	6.40E-21	88.05
	rs72863428	C	T	-0.141	0.015	7.46E-20	83.19
	rs77431062	A	G	0.242	0.027	1.37E-18	77.43
	rs186609898	A	G	-0.215	0.027	8.88E-16	64.66
	rs1880479	T	G	0.288	0.040	9.15E-13	51.02
	rs55935421	G	A	-0.210	0.030	1.20E-12	50.49
	rs10510718	T	C	0.199	0.028	1.82E-12	49.67
	rs11564447	G	T	-0.234	0.034	3.65E-12	48.3
	rs112763222	C	T	-0.239	0.037	8.89E-11	42.05
	rs9873086	G	A	-0.079	0.012	1.15E-10	41.55
	rs141269676	C	T	-0.226	0.036	4.93E-10	38.7
	rs147653845	A	G	0.236	0.038	5.77E-10	38.4
	rs113540515	G	C	-0.125	0.021	1.93E-09	36.05
	rs62258384	A	G	0.270	0.045	2.15E-09	35.84
	rs62258047	A	G	-0.109	0.019	6.41E-09	33.7
	rs111555748	G	A	-0.200	0.036	2.18E-08	31.33
RAD50	rs55730162	A	G	-0.231	0.014	1.21E-63	283.64
	rs143331722	A	G	-0.398	0.042	3.06E-21	89.5
	rs2070729	A	C	0.106	0.012	5.90E-19	79.11
	rs72795178	G	A	-0.299	0.037	7.64E-16	64.96
	rs35648164	T	C	-0.355	0.050	1.75E-12	49.75
	rs34362950	A	G	0.151	0.022	8.10E-12	46.74
	rs17690923	C	T	-0.088	0.013	6.14E-11	42.78
	rs116765892	A	G	-0.162	0.028	5.82E-09	33.89
FGF2	rs308403	T	C	-0.181	0.013	1.97E-45	200.12
	rs1960669	A	C	-0.124	0.017	4.58E-13	52.38
	rs13117763	A	G	-0.159	0.024	5.05E-11	43.16
	rs6419221	C	T	0.074	0.012	3.00E-09	35.18
	rs45513195	A	G	-0.224	0.041	3.20E-08	30.58
VIM	rs165530	C	A	0.242	0.013	2.85E-73	327.83
	rs2932912	T	G	0.178	0.012	6.92E-48	211.37
	rs7914640	A	G	0.195	0.017	1.27E-29	127.77
	rs71495253	T	C	0.195	0.022	3.72E-19	80.02
	rs11254295	G	T	-0.187	0.021	1.18E-18	77.74
	rs10774624	A	G	0.088	0.012	1.28E-13	54.88
	rs55793580	T	C	-0.116	0.016	2.08E-13	53.92

rs1687697	G	A	0.137	0.019	3.84E-13	52.72
rs4748343	A	G	0.099	0.015	9.41E-12	46.45
rs72775098	T	C	-0.264	0.039	1.42E-11	45.64
rs72775072	G	T	-0.179	0.027	2.13E-11	44.84
rs1375493	A	G	0.076	0.012	2.16E-10	40.31
rs11254387	C	G	-0.111	0.018	3.91E-10	39.15
rs7085551	A	G	0.282	0.048	5.10E-09	34.15
rs11254375	G	T	-0.074	0.013	8.08E-09	33.25

A1: effect allele; A2: other allele.

Supplementary Table S3. MR analysis demonstrating the association of target genes use with spontaneous abortion

	Methods	SNP	OR	LCI	UCI	P	Heterogeneity P	Egger intercept P	MR-PRESSO P
CTNNB1	IVW	20	0.99	0.96	1.02	0.604	0.627	0.707	0.662
	MR Egger	20	1.00	0.95	1.06	0.959			
	WM	20	0.99	0.96	1.03	0.714			
RAD50	IVW	8	1.08	1.01	1.15	0.019	0.580	0.345	0.559
	MR Egger	8	1.01	0.88	1.16	0.895			
	WM	8	1.04	0.96	1.12	0.361			
FGF2	IVW	5	1.21	1.11	1.33	4.69E-05	0.897	0.783	0.912
	MR Egger	5	1.17	0.88	1.54	0.356			
	WM	5	1.23	1.11	1.37	1.18E-04			
VIM	IVW	15	0.96	0.92	1.01	0.100	0.836	0.631	0.848
	MR Egger	15	0.94	0.83	1.05	0.290			
	WM	15	0.94	0.89	1.00	0.065			

MR: Mendelian randomization; OR: odds ratio; LCI: lower confidence interval; UCI: upper confidence interval; IVW: inverse variance weighted; WM: weighted median.

Supplementary Table S4. Leave-one-out sensitivity analysis for the MR results

	SNP	beta	se	P
RAD50	rs116765892	0.064	0.032	0.045
	rs143331722	0.082	0.035	0.019
	rs17690923	0.066	0.033	0.042
	rs2070729	0.071	0.034	0.034
	rs34362950	0.073	0.033	0.026
	rs35648164	0.073	0.032	0.021
	rs55730162	0.112	0.041	0.006
	rs72795178	0.063	0.034	0.061
	All	0.074	0.032	0.019
FGF2	rs13117763	0.191	0.051	1.64E-04
	rs1960669	0.213	0.053	5.42E-05

rs308403	0.176	0.070	0.012
rs45513195	0.196	0.049	5.65E-05
rs6419221	0.183	0.050	2.40E-04
All	0.193	0.047	4.69E-05

Supplementary Figure S1. Venn diagram showing the overlap of nicotine target genes across multiple databases

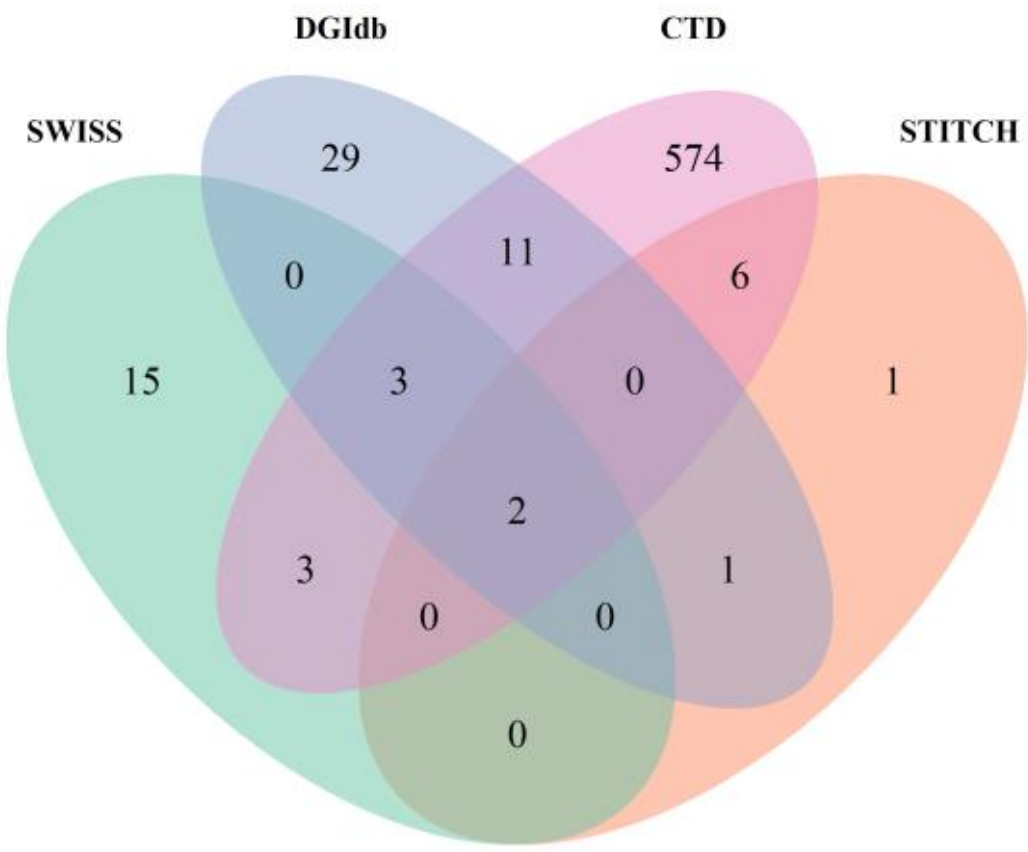

Venn diagram showing the overlap of nicotine target genes across multiple databases. Each circle represents one database, and overlapping regions indicate genes identified by multiple databases.

Supplementary Figure S2. Volcano plot of differentially expressed genes between spontaneous abortion and normal-pregnancy controls

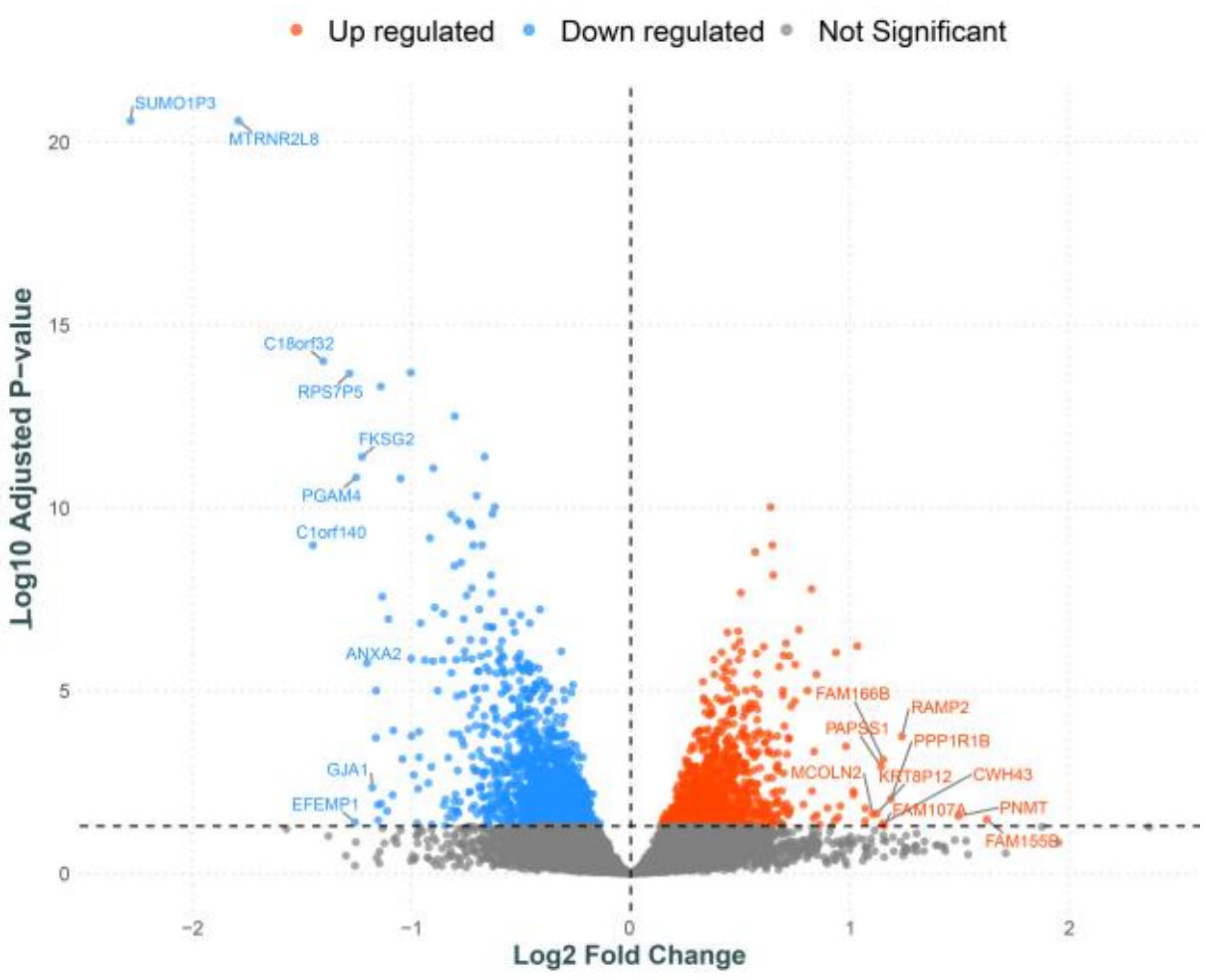

Red dots represent significantly upregulated genes, blue dots represent significantly downregulated genes (adjusted P < 0.05), and gray dots represent genes with no significant differential expression.

Supplementary Figure S3. Heatmap depicting the expression profiles of the differentially expressed genes

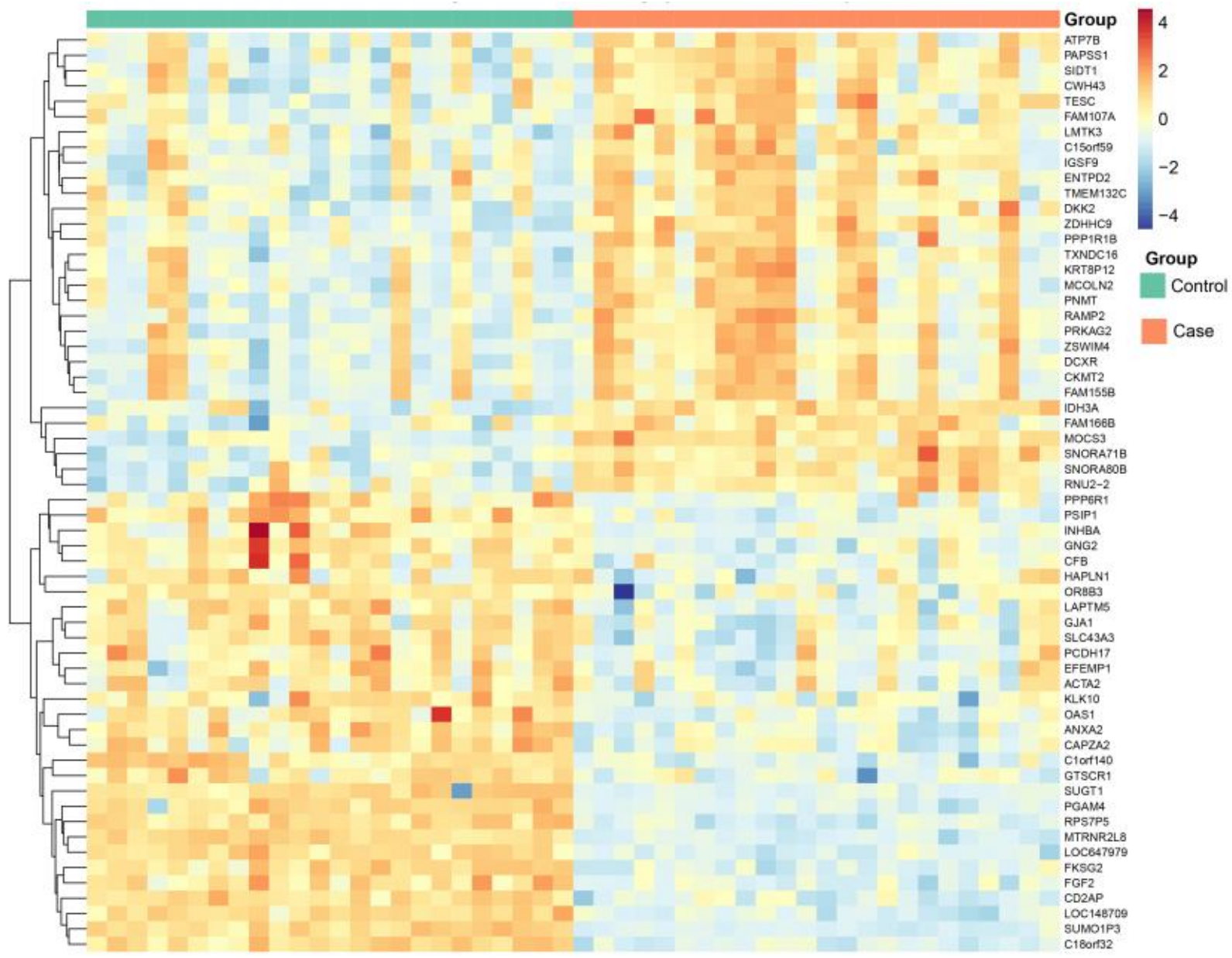

Supplementary Figure S4. Venn diagram integrating nicotine targets with SA-related DEGs.

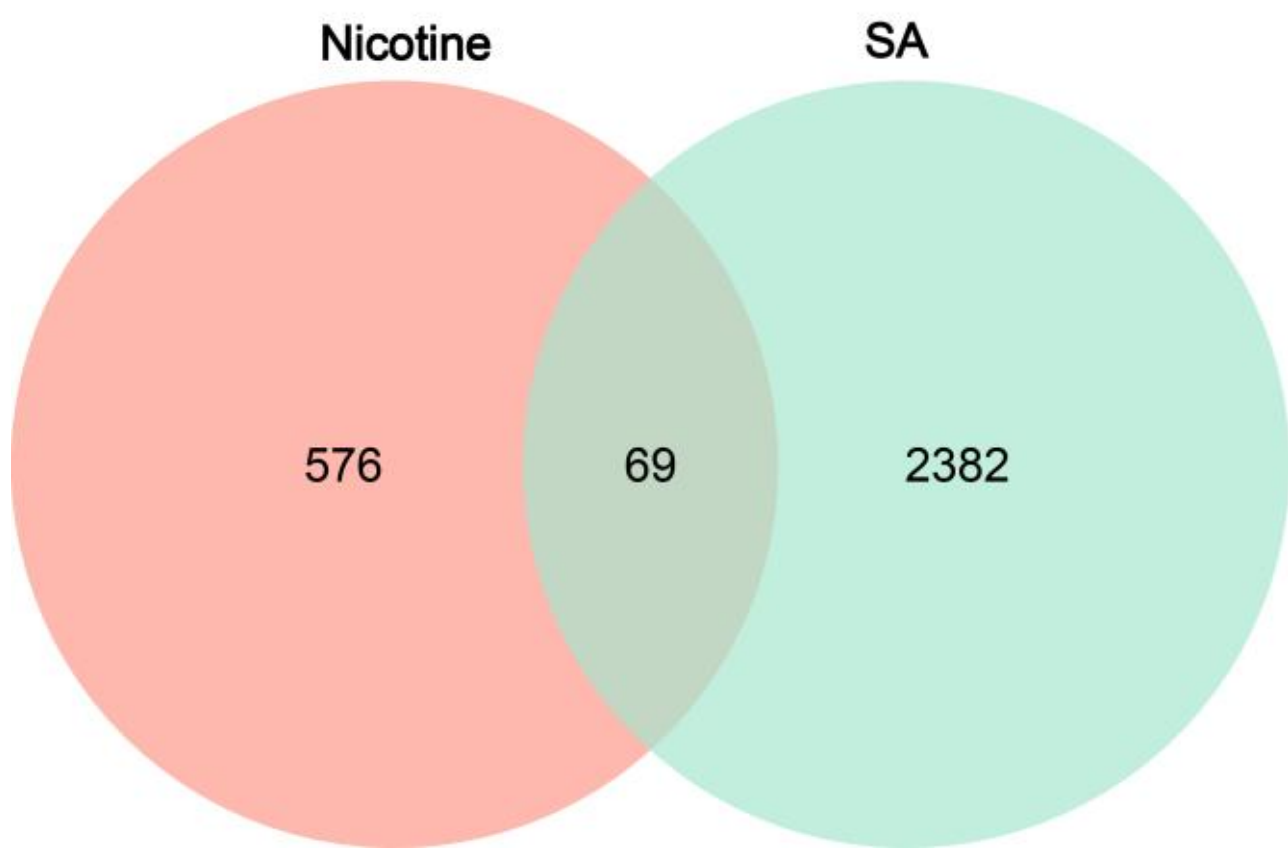

DEGs: differentially expressed genes; SA: spontaneous abortion.

Supplementary Figure S5. Compound–target–disease network linking nicotine and spontaneous abortion

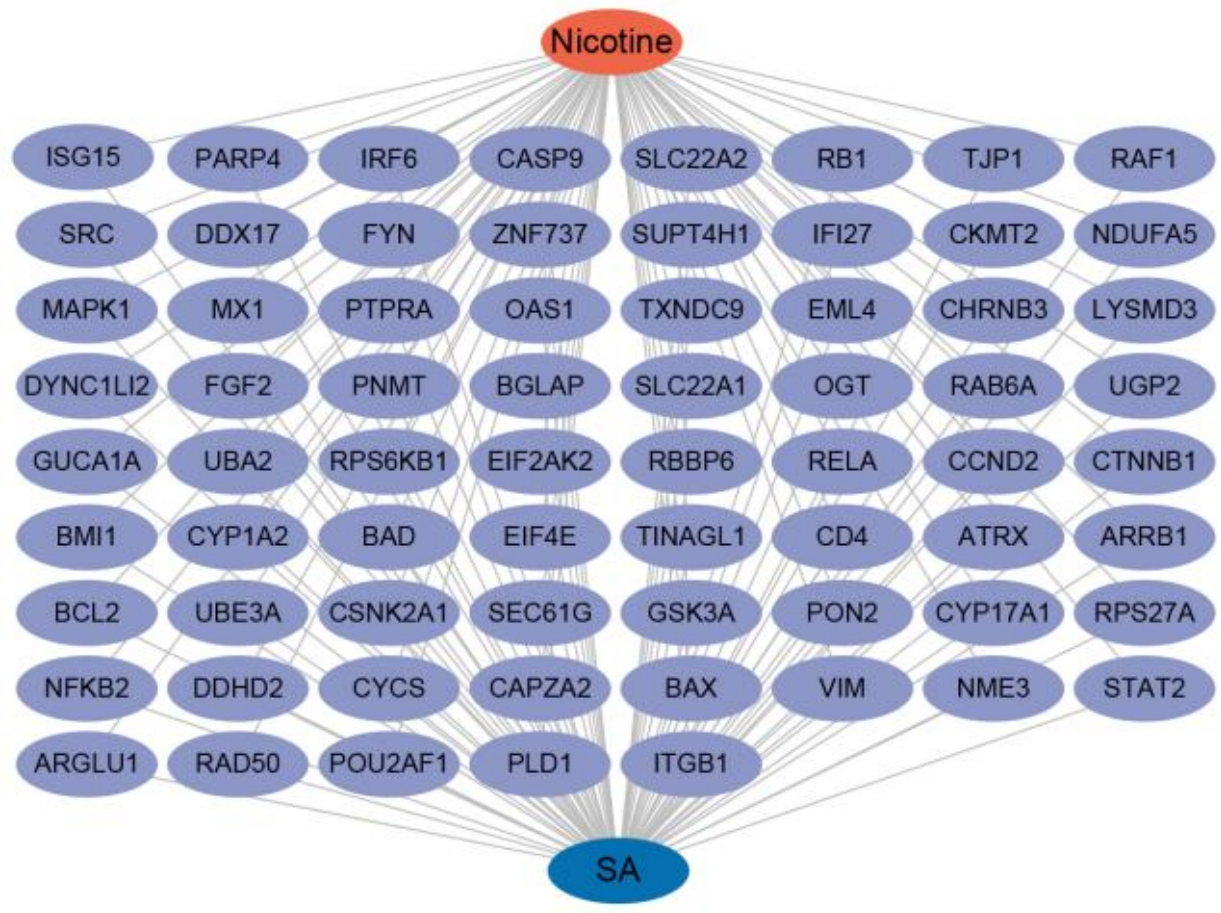

Supplementary Figure S6. GO functional enrichment analysis for the candidate genes

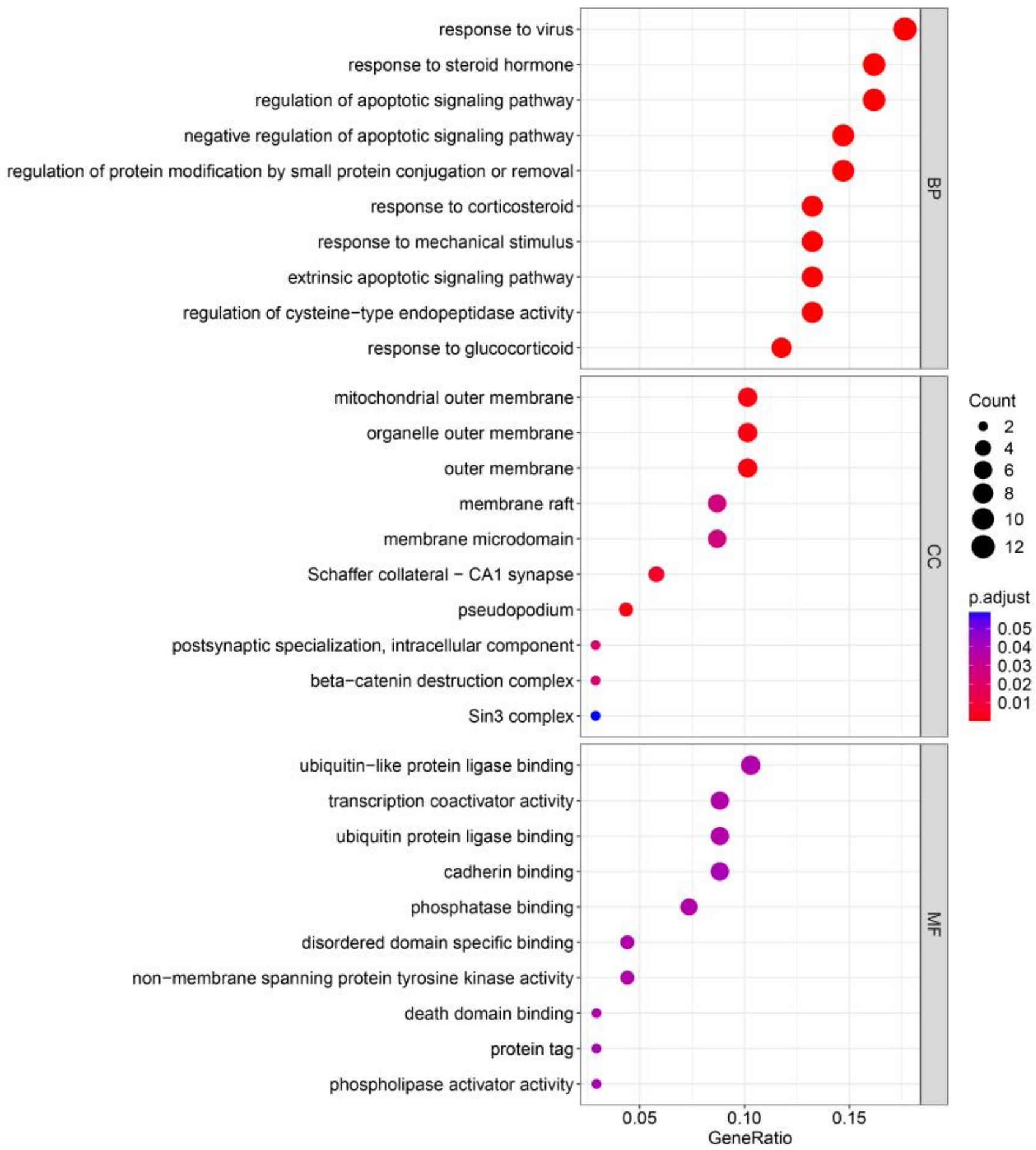

GO: gene ontology; BP: biological process; CC: cellular component; MF: molecular function.

Supplementary Figure S7. KEGG pathway analysis for the candidate genes

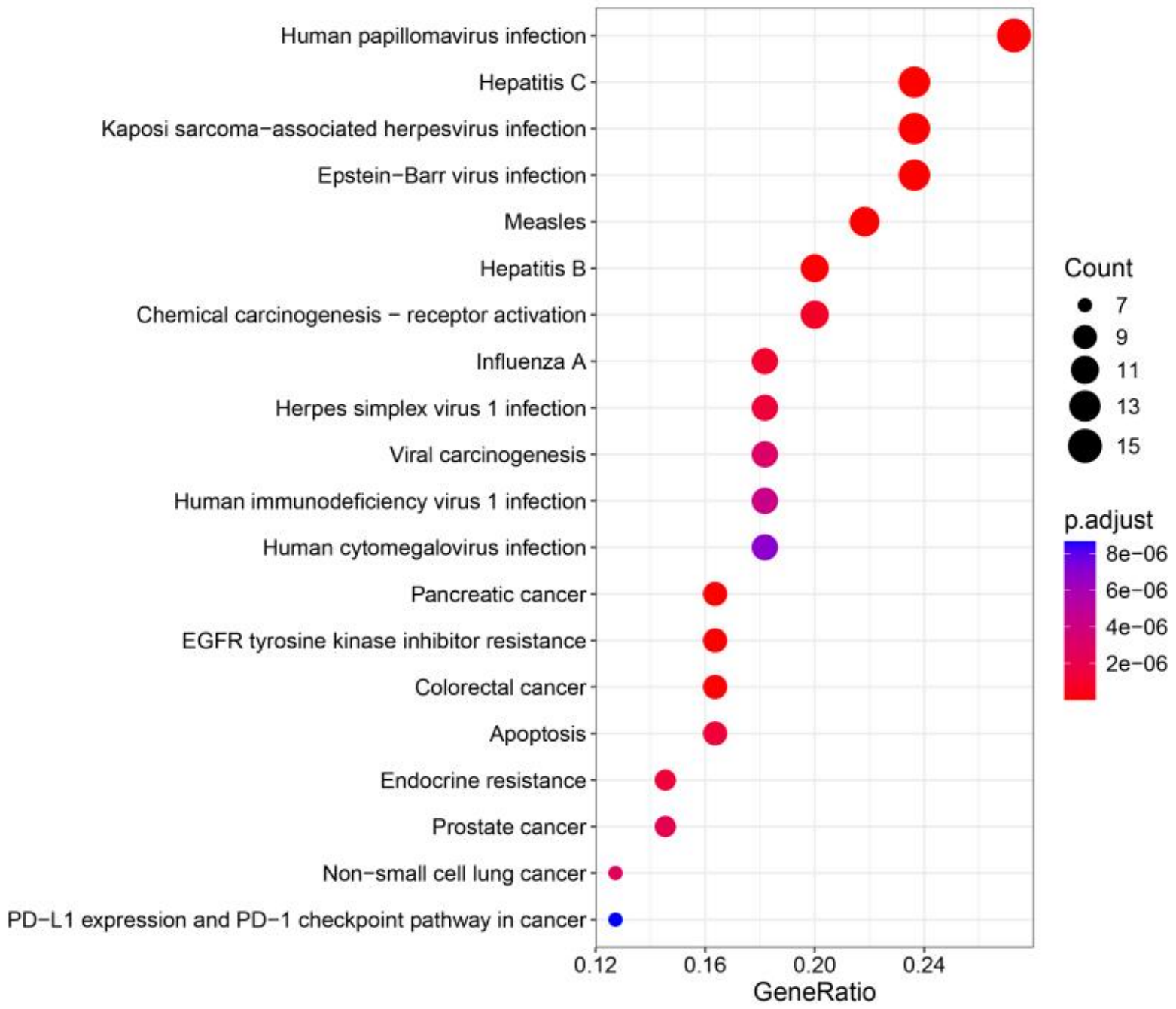

KEGG: Kyoto Encyclopedia of Genes and Genomes.

Supplementary Figure S8. Binomial deviance of the LASSO model as a function of the regularization parameter λ

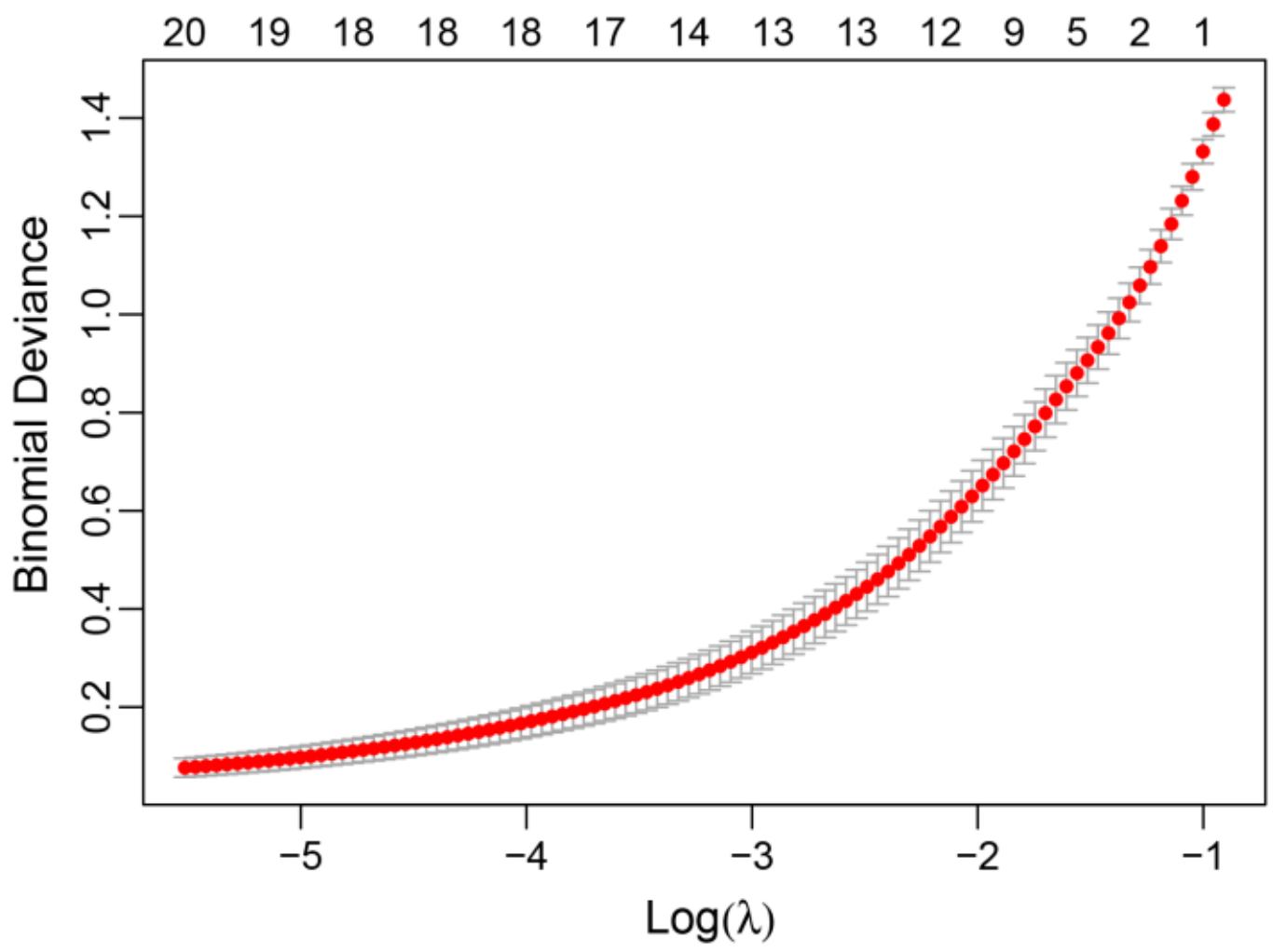

LASSO: least absolute shrinkage and selection operator.

Supplementary Figure S9. LASSO coefficient paths of gene candidates with changing log λ

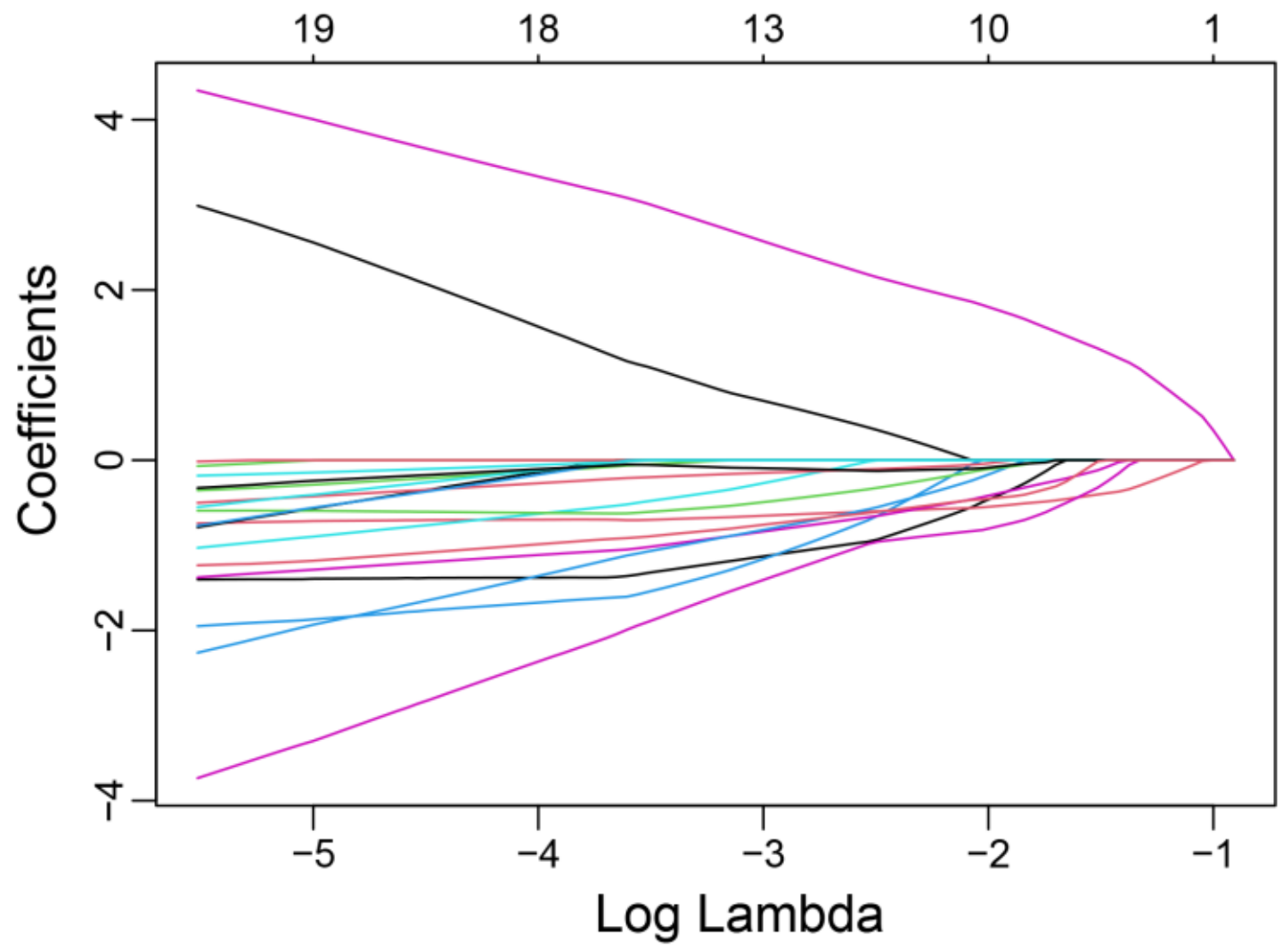

LASSO: least absolute shrinkage and selection operator.

Supplementary Figure S10. Cross-validation accuracy versus number of features for SVM-RFE

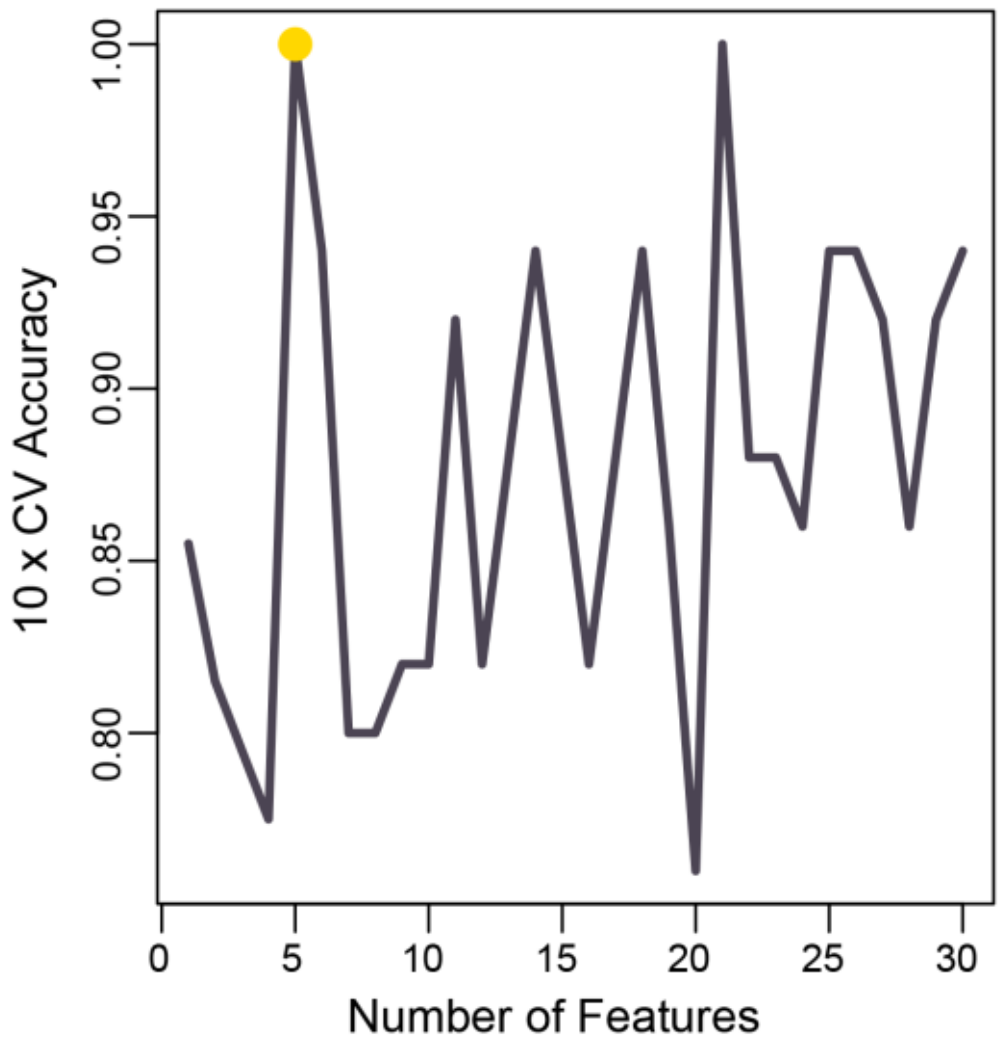

SVM-RFE: support vector machine–recursive feature elimination.

Supplementary Figure S11. Venn diagram summarizing genes selected by LASSO and SVM-RFE

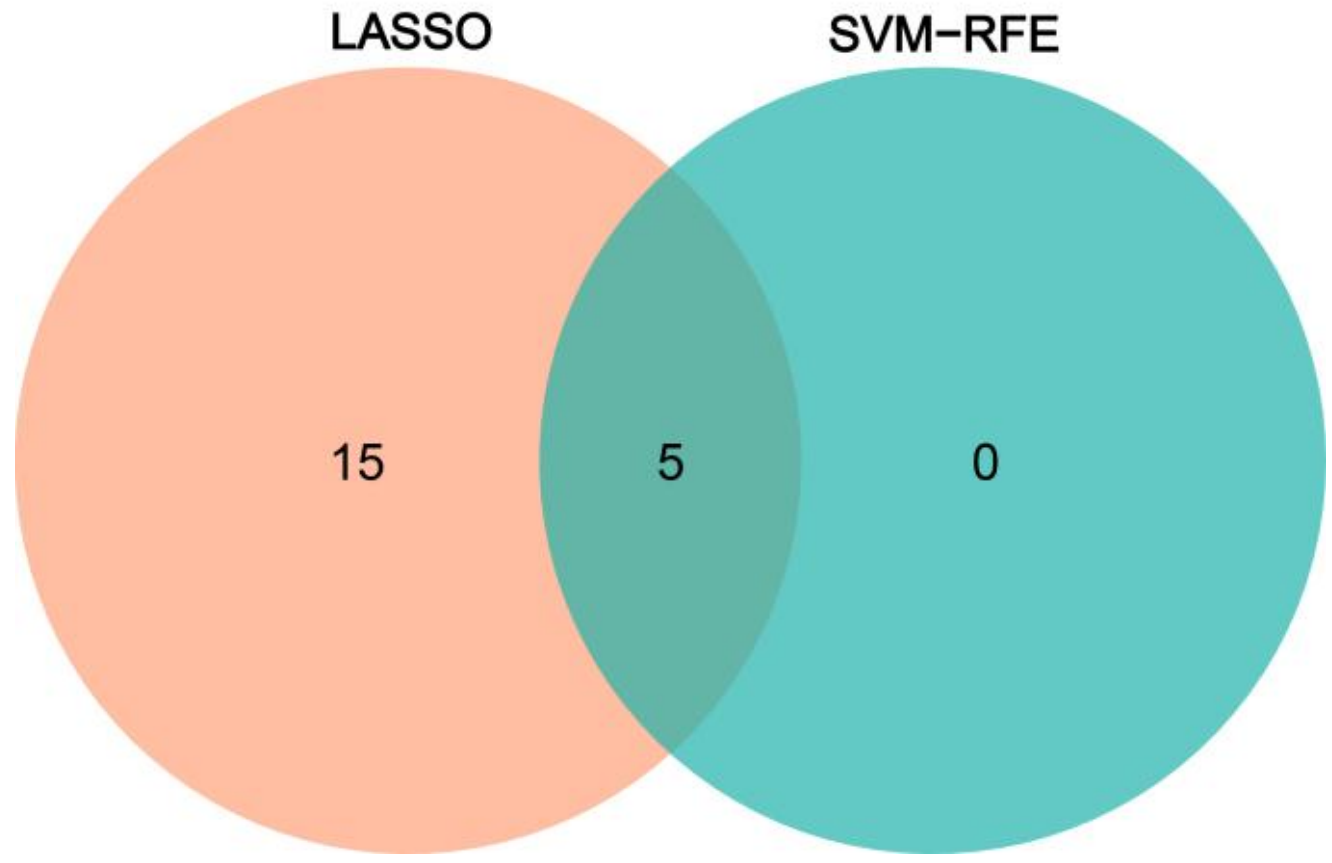

LASSO: least absolute shrinkage and selection operator; SVM-RFE: support vector machine–recursive feature elimination.

Supplementary Figure S12. Violin plots comparing expression levels of the five hub genes between control and spontaneous abortion groups

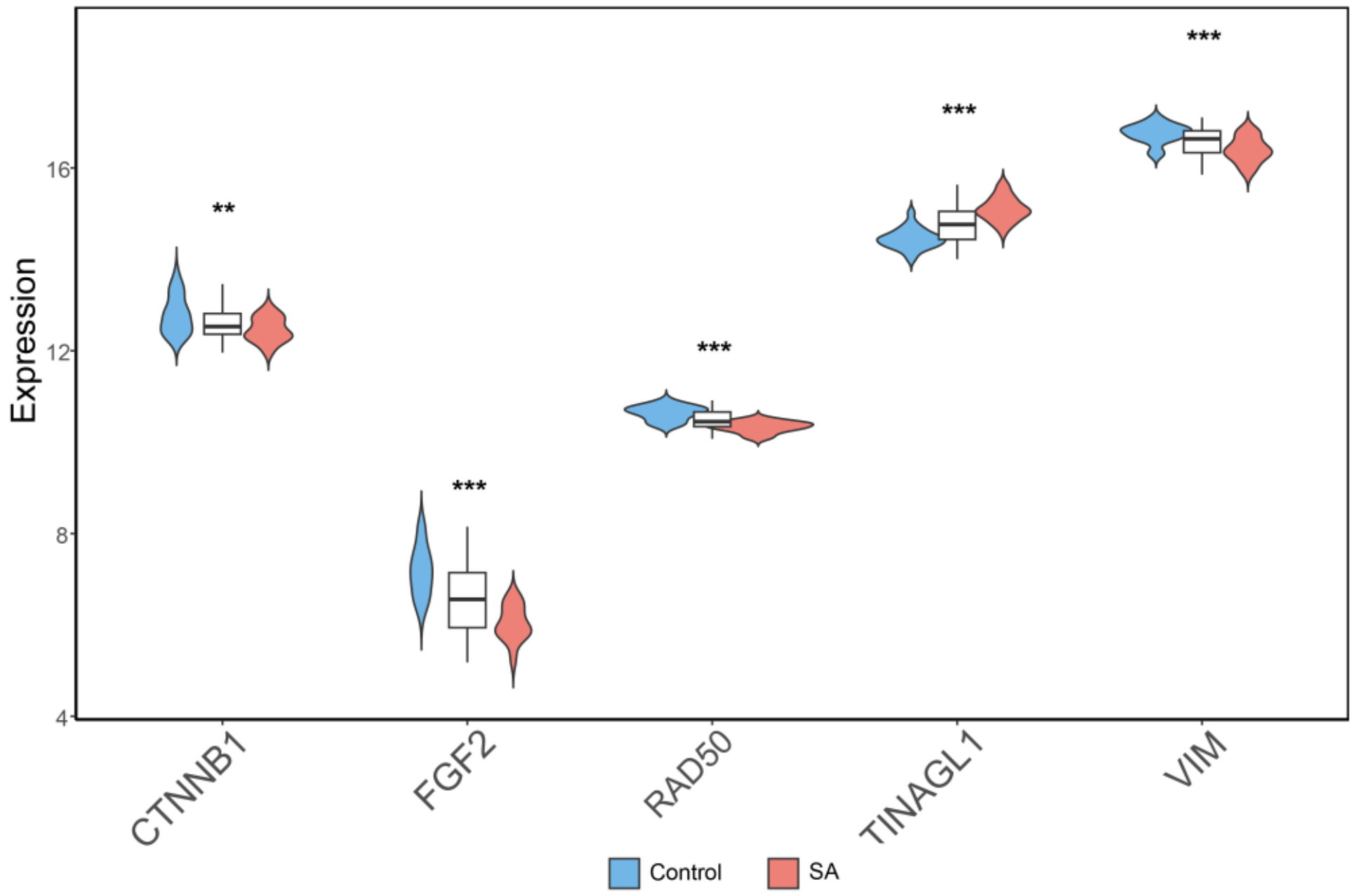

SA: spontaneous abortion; **P < 0.01, ***P < 0.001.

Supplementary Figure S13. ROC curves of the five candidate hub genes for diagnostic evaluation

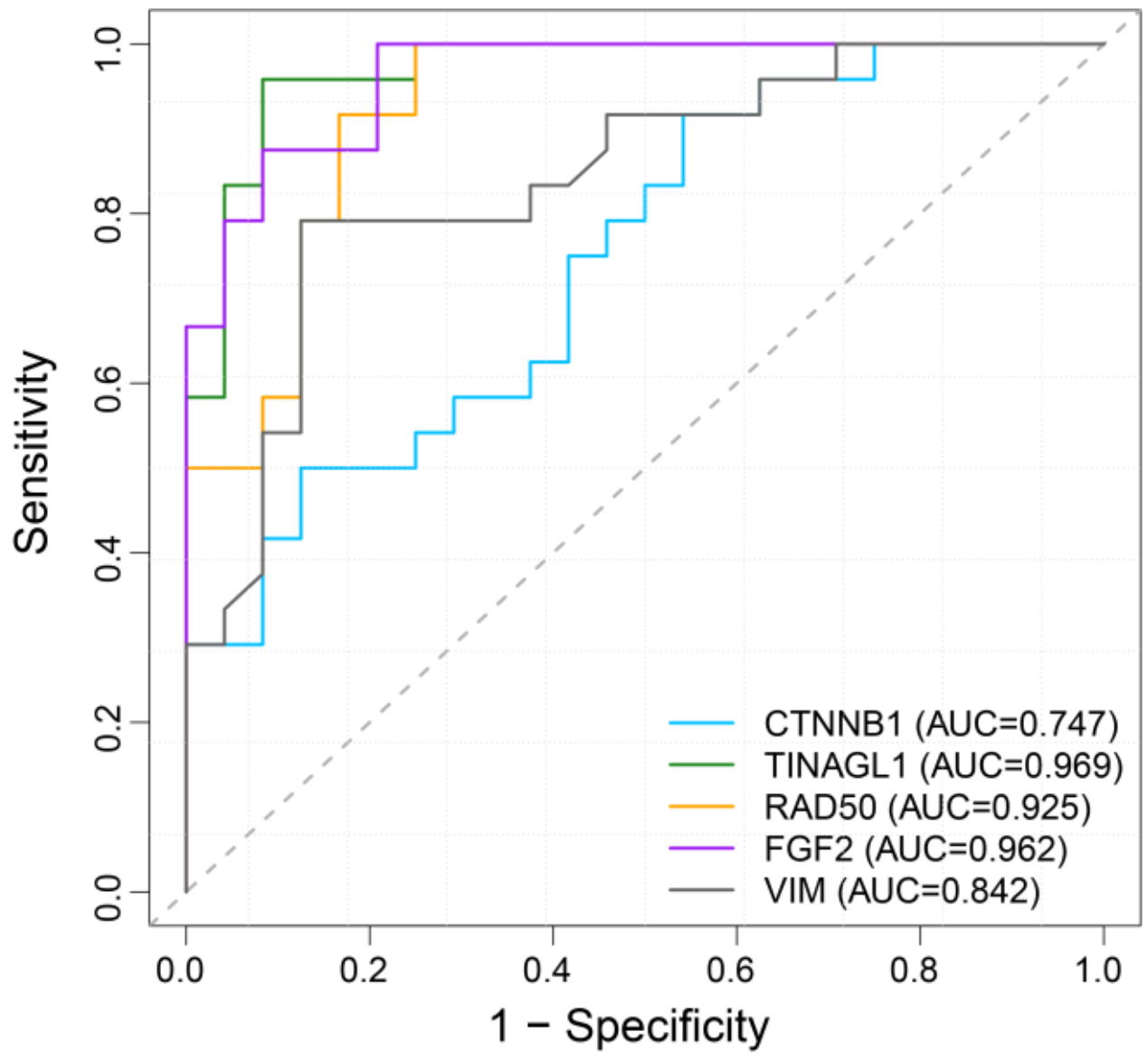

ROC: receiver operating characteristic; AUC: area under the curve.

Supplementary Figure S14. Stacked bar plot showing the relative fractions of immune-cell subsets in each sample

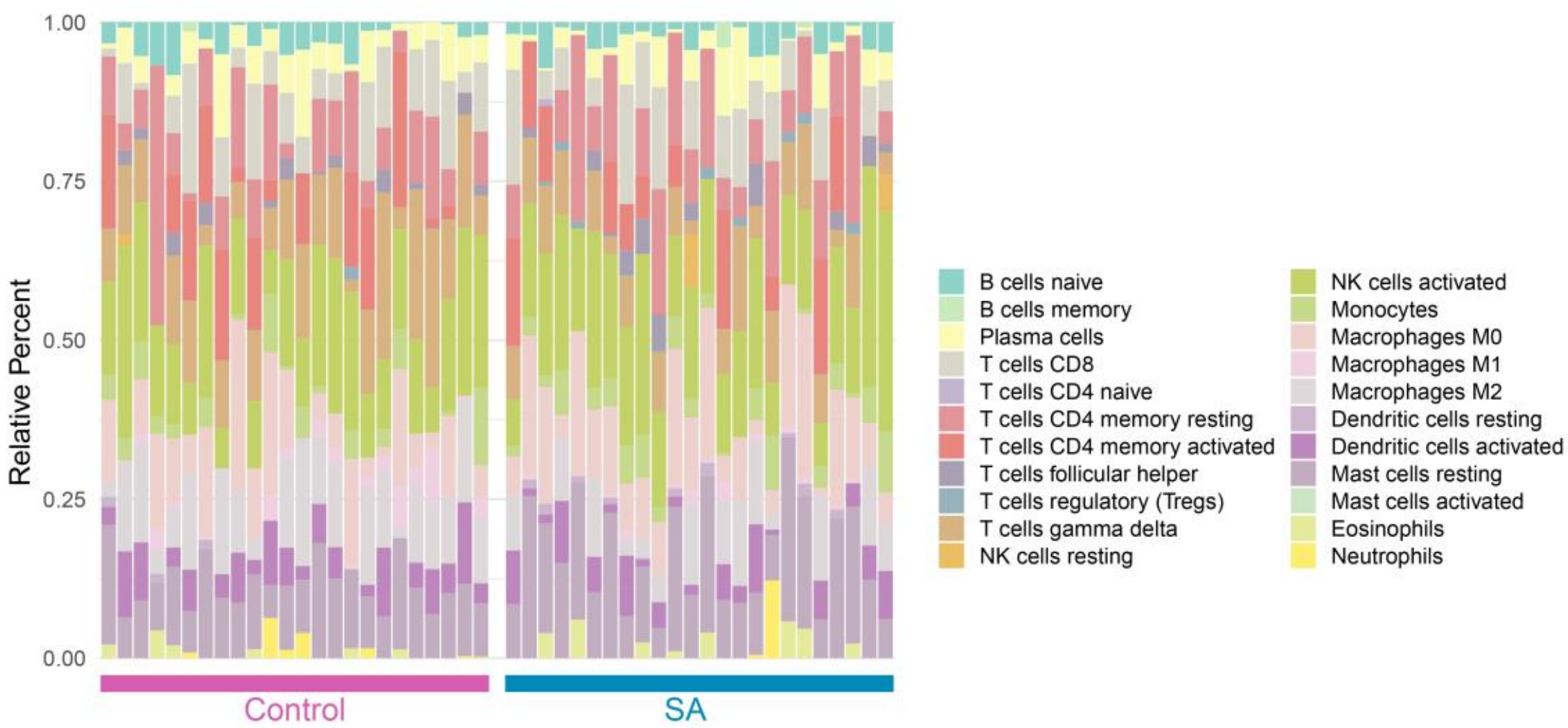

SA: spontaneous abortion.

Supplementary Figure S15. Spearman correlation heatmap of pairwise relationships among the immune-cell subsets

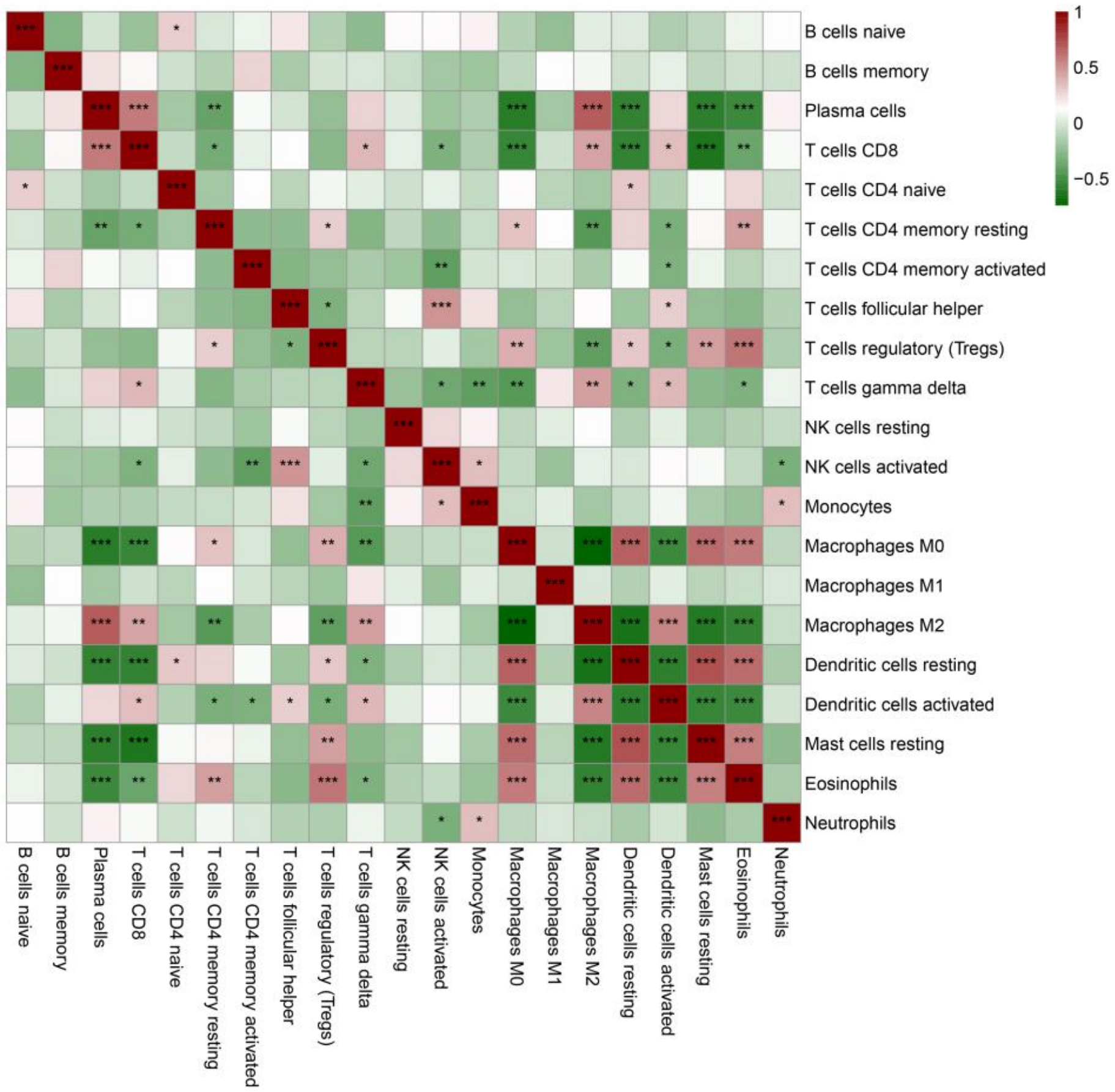

Spearman correlation heatmap of pairwise relationships among the immune-cell subsets. Data were derived from the GSE165004 dataset using CIBERSORTx. The heatmap shows pairwise Spearman correlation coefficients between immune-cell subsets. Red indicates positive correlation, blue indicates negative correlation. *P < 0.05, **P < 0.01, ***P < 0.001.

Supplementary Figure S16. Two-sample MR forest plot of the causal effects of target genes on spontaneous abortion

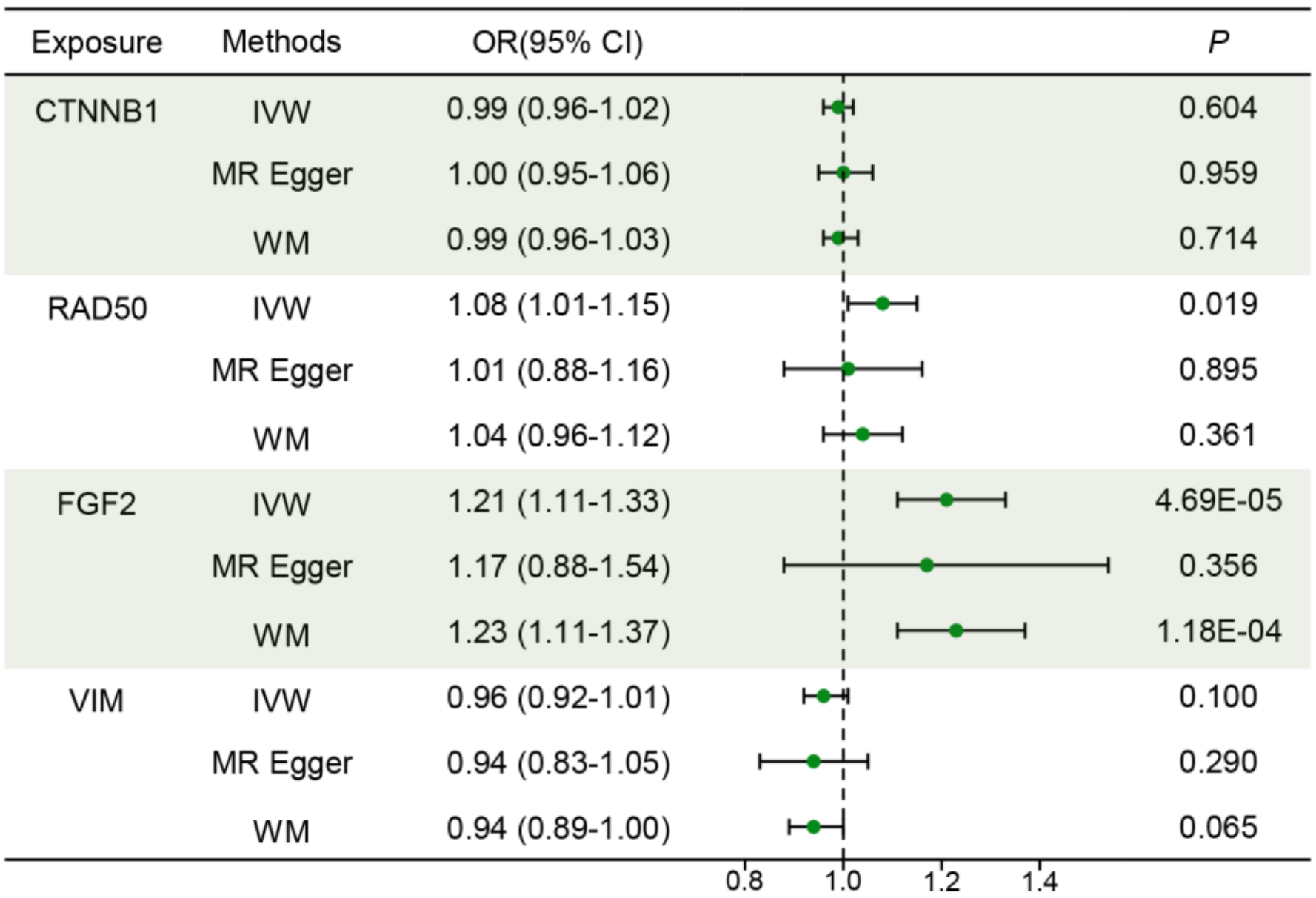

Exposure	Methods	OR(95% CI)	*P*
CTNNB1	IVW	0.99 (0.96-1.02)	0.604
	MR Egger	1.00 (0.95-1.06)	0.959
	WM	0.99 (0.96-1.03)	0.714
RAD50	IVW	1.08 (1.01-1.15)	0.019
	MR Egger	1.01 (0.88-1.16)	0.895
	WM	1.04 (0.96-1.12)	0.361
FGF2	IVW	1.21 (1.11-1.33)	4.69E-05
	MR Egger	1.17 (0.88-1.54)	0.356
	WM	1.23 (1.11-1.37)	1.18E-04
VIM	IVW	0.96 (0.92-1.01)	0.100
	MR Egger	0.94 (0.83-1.05)	0.290
	WM	0.94 (0.89-1.00)	0.065

MR: Mendelian randomization; OR: odds ratios; CI: confidence intervals; IVW: inverse variance weighted; WM: weighted median. SA: spontaneous abortion.

Supplementary Figure S17. t-SNE plot showing the cellular composition of decidual tissue

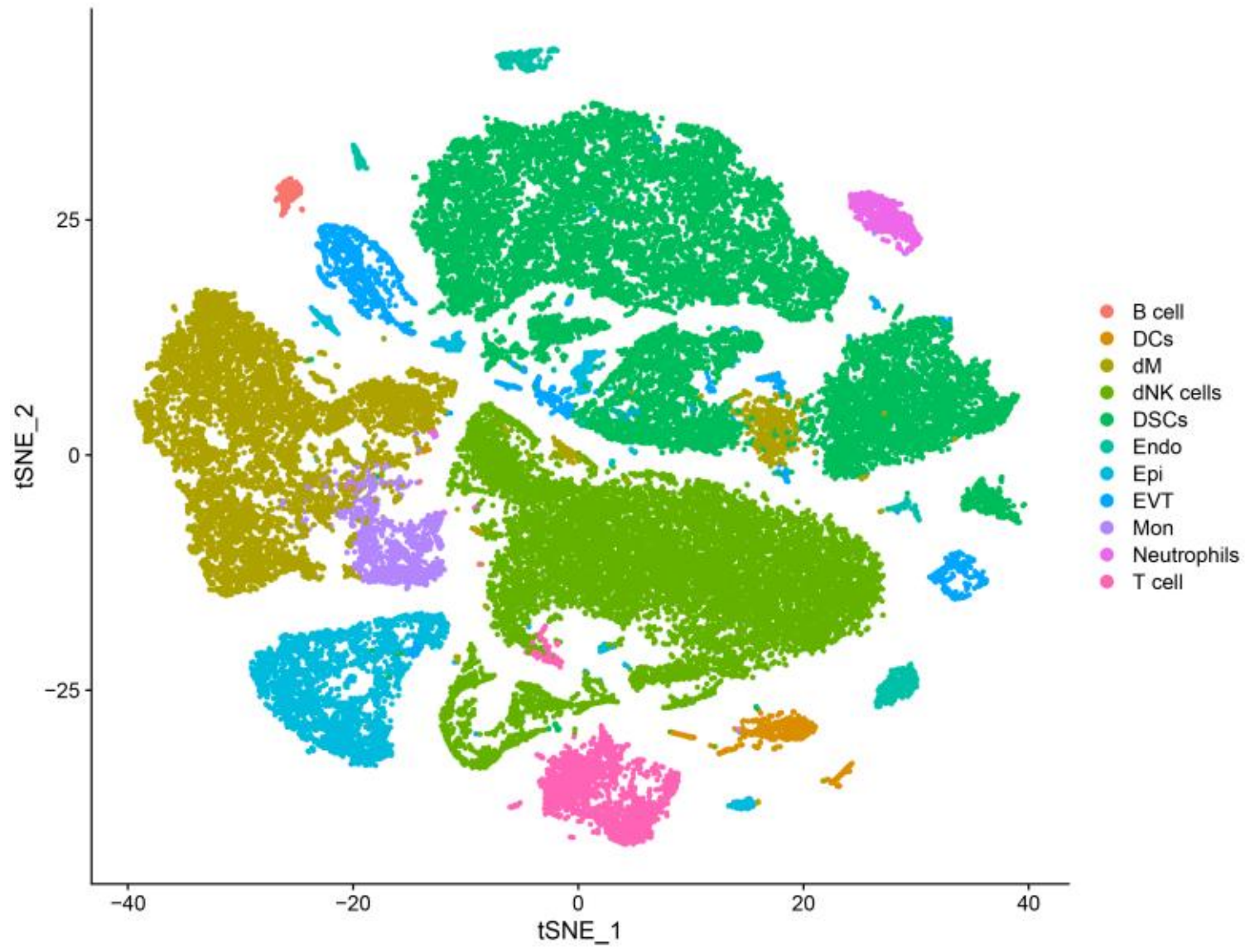

Eleven cell types were identified: B cells, T cells, dendritic cells (DCs), decidual NK cells (dNKs), decidual macrophages (dM), monocytes (Mon), neutrophils, decidual stromal cells (DSCs), endothelial cells (Endo), epithelial cells (Epi), and extravillous trophoblasts (EVT). t-SNE: t-distributed stochastic neighbor embedding.

Supplementary Figure S18. Dot plot of expression levels and percentage of expressing cells for the five hub genes across different cell types

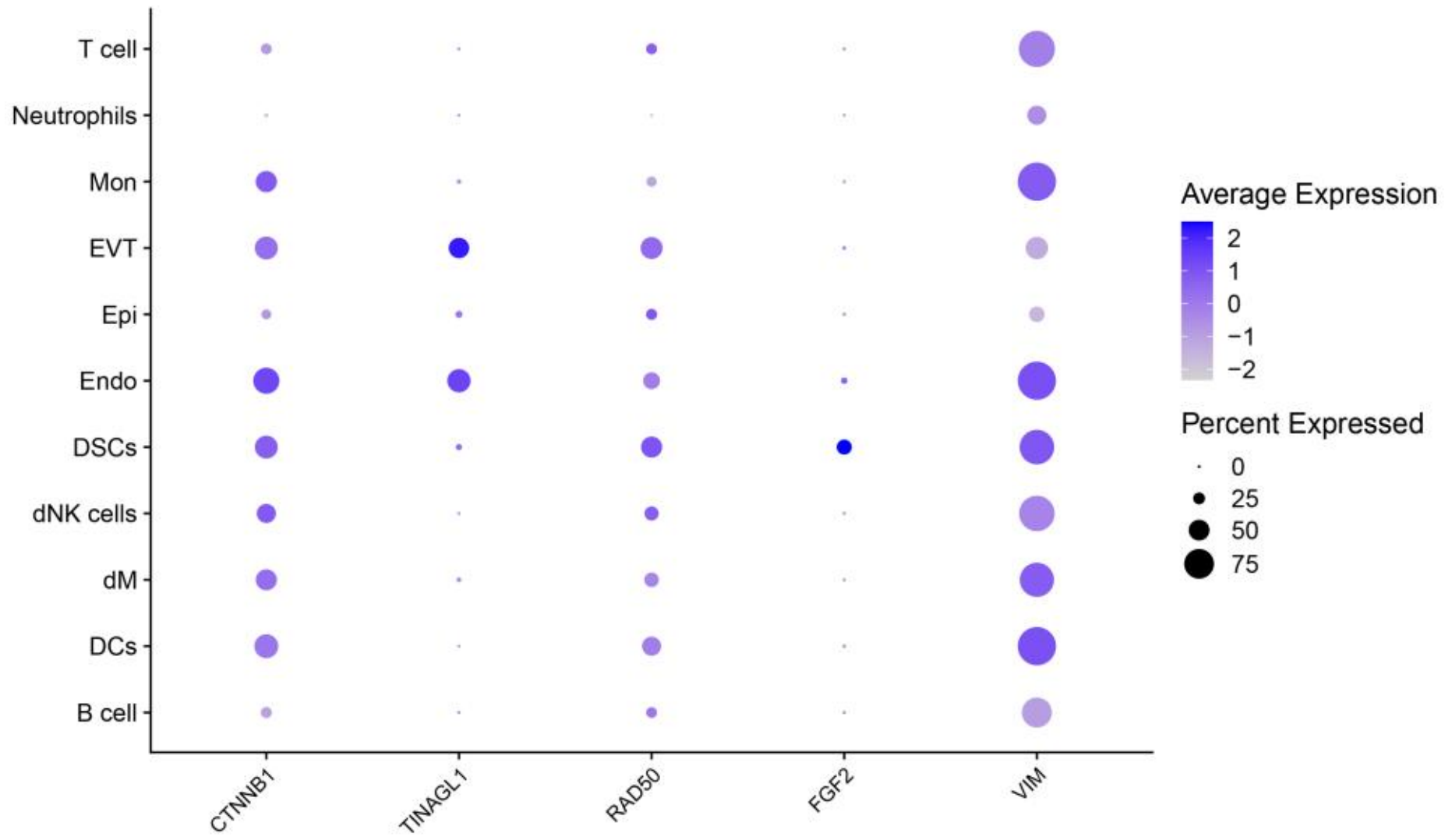

DCs: dendritic cells; dNKs: decidual NK cells; dM: decidual macrophages; Mon: monocytes; DSCs: decidual stromal cells; Endo: endothelial cells; Epi: epithelial cells; EVT: extravillous trophoblasts.

Supplementary Figure S19. Feature plots illustrating the spatial expression patterns of core target genes.

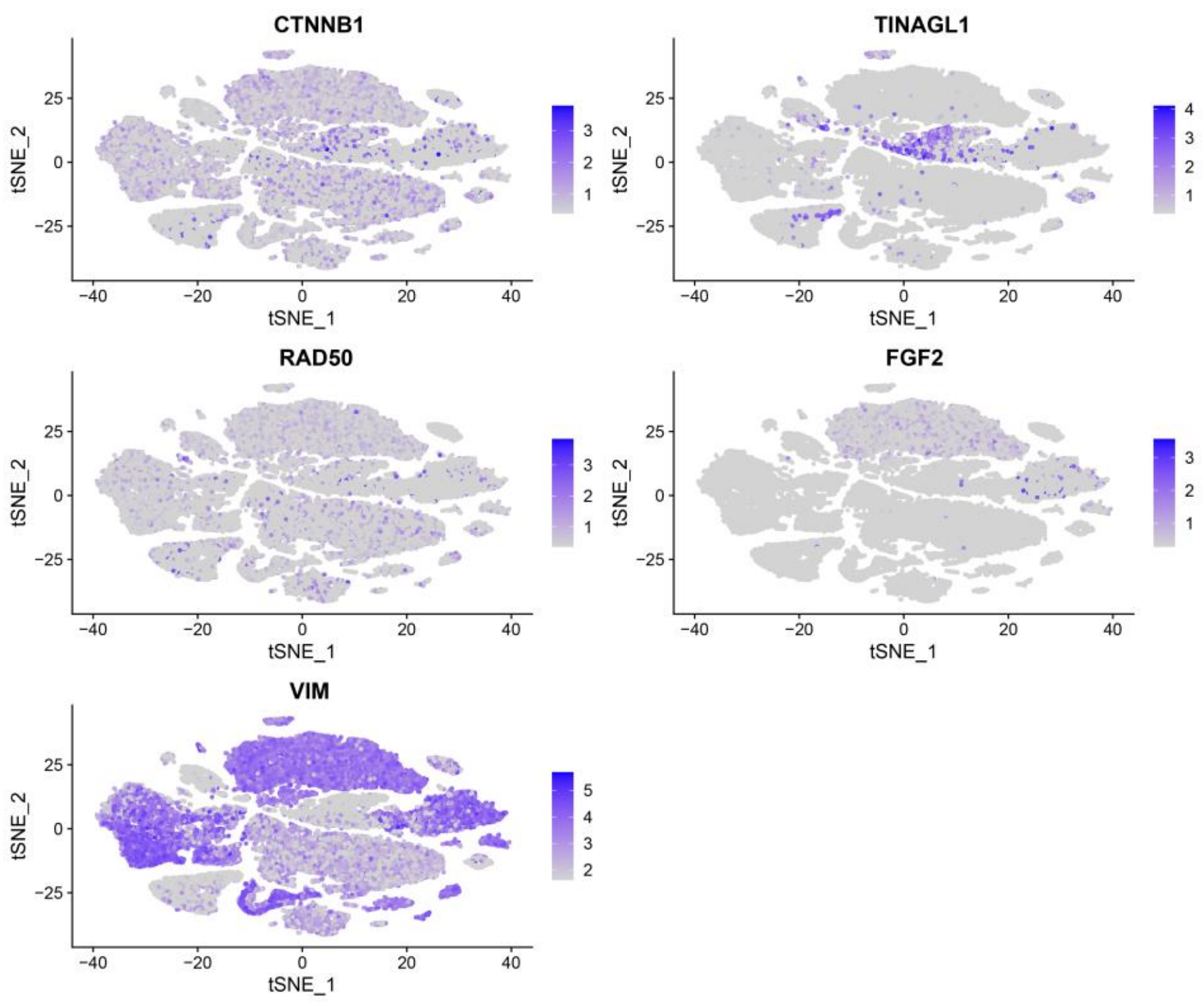

Supplementary Figure S20. Differential expression analysis of the five hub genes between SA and control groups in key cell types.

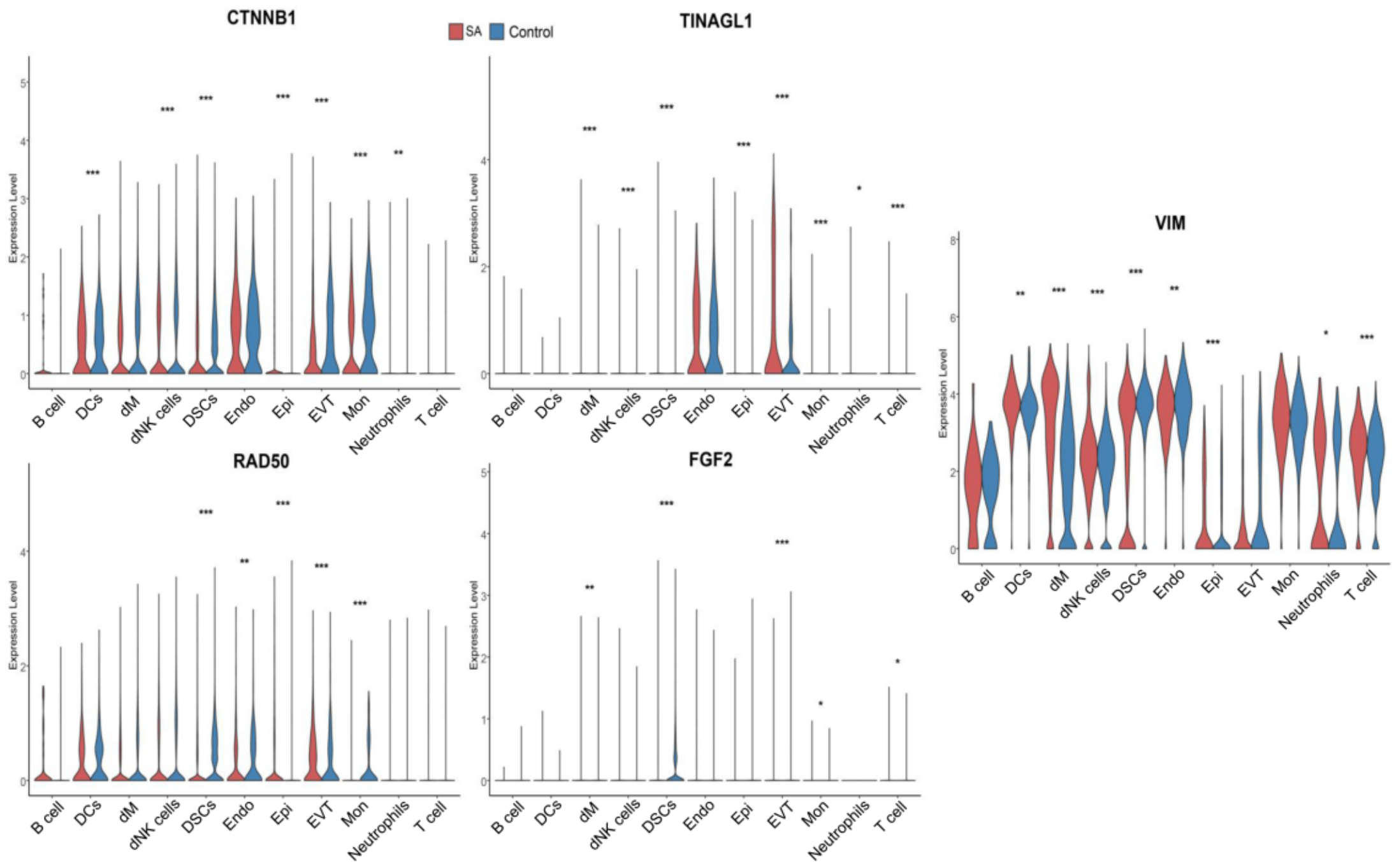

DCs: dendritic cells; dNKs: decidual NK cells; dM: decidual macrophages; Mon: monocytes; DSCs: decidual stromal cells; Endo: endothelial cells; Epi: epithelial cells; EVT: extravillous trophoblasts; SA: spontaneous abortion. *P < 0.05, **P < 0.01, ***P < 0.001.

Supplementary Figure S21. Molecular docking of nicotine with the five core target proteins

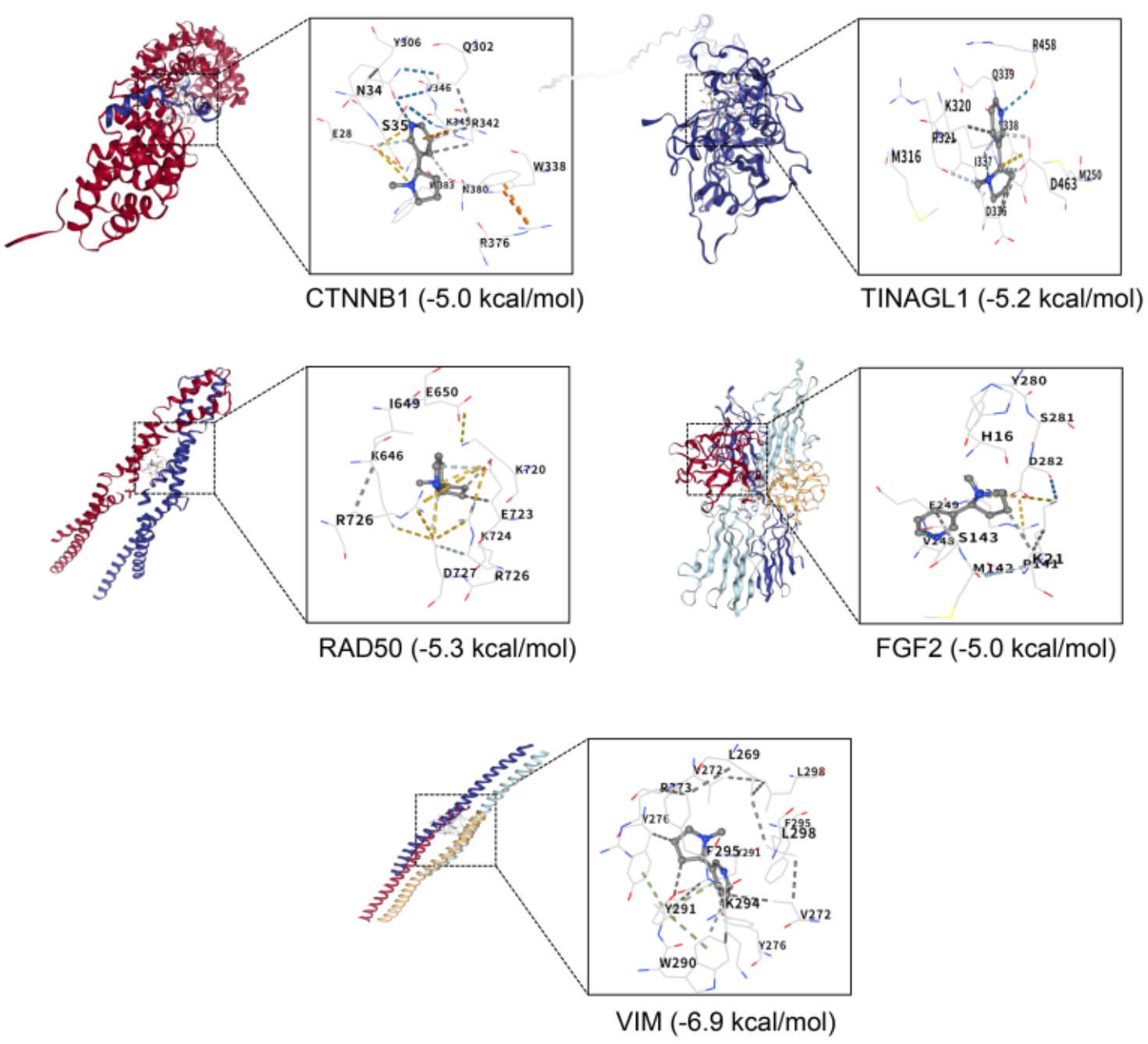

Supplement: Supplementary file 1 [file TID-24-174-s1.pdf]
